# Synthesis and Characterization of Octacyano-Fe-Phthalocyanine

**DOI:** 10.1021/acsomega.3c02638

**Published:** 2023-07-17

**Authors:** Momoka Isobe, Shota Nakayama, Shunsuke Takagi, Kakeru Araki, Kaname Kanai

**Affiliations:** Department of Physics and Astronomy, Faculty of Science and Technology, Tokyo University of Science, 2641 Yamazaki, Noda, Chiba 278-8510, Japan

## Abstract

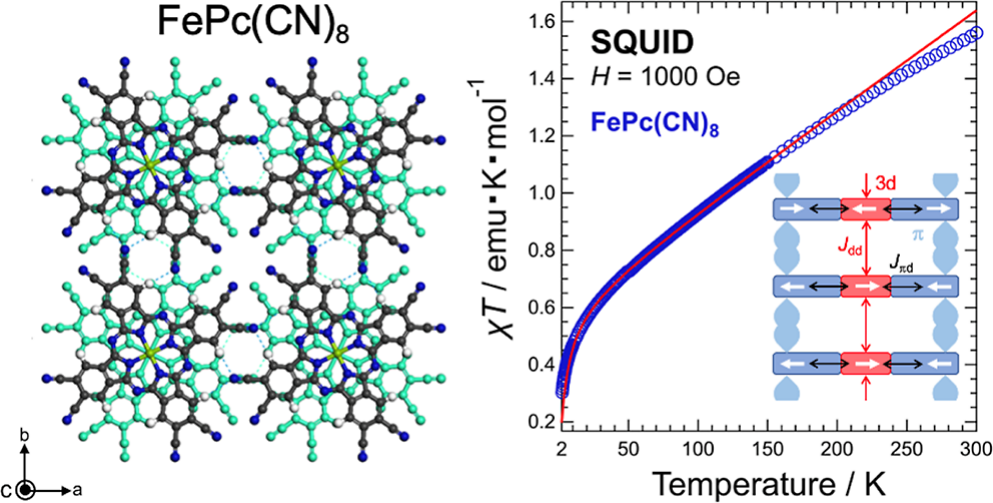

Octacyano-metal-phthalocyanine MPc(CN)_8_ is a promising n-type stable organic semiconductor material with eight cyano groups, including a strong electron-withdrawing group at its molecular terminals. However, a thorough investigation of MPc(CN)_8_ has not yet been conducted. Therefore, we synthesized FePc(CN)_8_ and investigated its crystal structure, chemical and electronic states, electrical properties, photocatalytic activity, and magnetic properties. In this paper, we discuss the various properties of MPc(CN)_8_ in comparison with those of FePc. X-ray diffraction measurements indicated that the crystal structure of FePc(CN)_8_ was strongly influenced by the cyano groups and differed from the α- and β-forms of FePc. The space group *P*4/*mcc* structure of FePc(CN)_8_ was similar to that of the x-form of LiPc. The ultraviolet–visible (UV–vis) absorption spectrum of FePc(CN)_8_ was observed at wavelengths longer than that of FePc. Density functional theory-based molecular orbital calculations indicated that the energy gap of FePc(CN)_8_ is smaller than that of FePc, which can lead to the observation of the Q-band in the UV–vis absorption spectrum of FePc(CN)_8_ at longer wavelengths than that of FePc. Because FePc(CN)_8_ has a wider optical absorption band in the visible region than FePc, its photocatalytic activity is approximately four times higher than that of FePc. The conductivity of FePc(CN)_8_ was also higher than that of FePc, which is due to the larger overlap of π-electron clouds of the molecules in the crystal structure of FePc(CN)_8_. Magnetic measurements revealed that FePc(CN)_8_ exists in an antiferromagnetic ground state. The magnetic properties of FePc(CN)_8_ are specific to its crystal structure, with direct exchange interactions between Fe^2+^ ions and π-electron-mediated interactions. In particular, the Pauli paramagnetic behavior at high temperatures and the antiferromagnetic behavior at low temperatures (Weiss temperature θ = −4.3 ± 0.1 K) are characteristic of the π–d system.

## 1 Introduction

**Figure 1:**
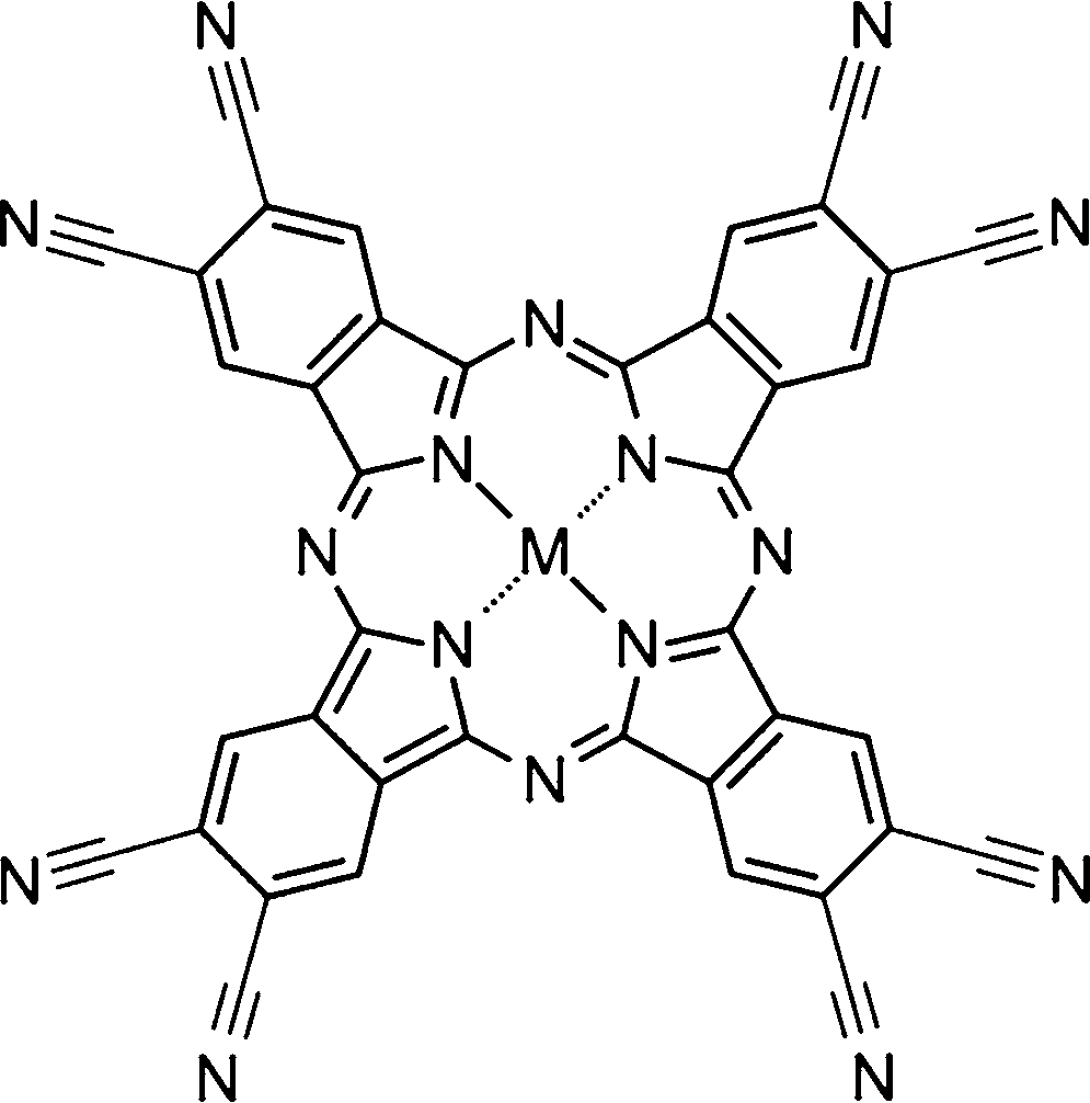
Molecular structure of octacyano-MPcs (MPc(CN)_8_s).

Metal-substituted phthalocyanines (MPc) (M = Li, Mn, Fe, Co, Ni, Cu, Zn, Sn, Pb...) are molecules with strong absorption in the ultraviolet–visible (UV–vis) region and constitute one of the representative organic semiconductor classes.^1−3^ Owing to their chemical stability, thermal stability, and ease of thin film formation, they are also expected to be applied to various devices, such as solar cells, organic light-emitting diodes, field-effect transistors, gas sensors, and storage devices.^4−8^ In recent years, efforts have been made to realize complementary logic circuits in organic semiconductors, and it is essential to identify materials that exhibit both p- and n-type conductivity. MPcs are a class of materials in which p- and n-type molecules can be produced with only minor chemical modifications. Although many MPcs exhibit p-type conductivity,^3,9,10^ they can be easily switched to n-type conductivity via chemical modification. In addition to the type of metal to be substituted, the chemical, electrical, and physical properties of MPcs can be controlled by replacing the end groups. For example, perfluoro-MPcs (F_16_MPcs), in which all terminal hydrogens are replaced by fluorine, have high electron affinity due to the strong electronegativity of fluorine, which reduces the energies of the molecular orbitals. F_16_MPcs are typical n-type organic semiconductors that are chemically stable in air.^6,11,12^ Octacyano-MPcs (MPc(CN)_8_s) (Figure 1), such as F_16_MPc, have a highly electronegative cyano group as the terminal group, making them n-type materials with large electronegativity. A very strong electronegative molecule similar to MPc(CN)_8_, octacyanotetrapyrazinoporphyrazine, has also been reported.^13^ MPc(CN)_8_ has been reported to be a precursor of poly-MPc (PMPc), which is a two-dimensional polymerized MPc framework.^14−16^ The polymerization process involves heating MPc(CN)_8_, which leads to the formation of polymer sheets through the reaction of the cyano groups at the ends of different MPc(CN)_8_ molecules that serve as binding sites. PMPcs have attracted considerable attention in recent years because of their potential as narrow-energy-gap semiconductors and new magnetic materials. Many existing two-dimensional materials, such as graphene, exhibit nonmagnetic properties. However, several PMPc containing transition metals have been theoretically predicted to exhibit ferromagnetism and antiferromagnetism, making them particularly promising as novel magnetic materials.^17^ Although there are previous reports on the synthesis of MPc(CN)_8_,^15,18,19^ the detailed properties of MPc(CN)_8_ have not yet been reported. The choice of n-type materials for organic semiconductors is limited because organic anion radicals are chemically unstable. Therefore, it is important to understand the properties of MPc(CN)_8_ as a prospective material for molecular designs and applications in organic optoelectronic devices.

In this paper, we report the synthesis and detailed characterization of octacyano-Fe-phthalocyanine, FePc(CN)_8_. The crystal structure of FePc(CN)_8_ was determined using X-ray diffraction (XRD), and its chemical state was investigated using Fourier transform infrared (FTIR) spectroscopy and X-ray photoelectron spectroscopy (XPS). The electronic states near the frontier orbitals were clarified using ultraviolet photoemission spectroscopy (UPS) combined with inverse photoemission spectroscopy (IPES). In addition, the electrical properties and photocatalytic activity of FePc(CN)_8_ were measured and compared with those of FePc. As a result, FePc(CN)_8_ showed superior electrical conductivity and photocatalytic activity compared to those of FePc. Furthermore, magnetic measurements revealed that FePc(CN)_8_ exhibits antiferromagnetic interaction below the Weiss temperature θ = −4.3 ± 0.1 K. The magnetism of FePc(CN)_8_ is caused by the coupling between the Fe 3d-orbitals as well as the coupling between the π-orbital of *Pc* and the Fe 3d-orbital, which is the typical behavior of π–d electron systems.

## 2 Experimental Section

### 2.1 Preparation of FePc(CN)_8_

For the synthesis of FePc(CN)_8_, 1,2,4,5-tetracyanobenzene (TCNB: Tokyo Chemical Industry Co. (TCI), T0988-5G, purity: >98%) and FeCl_2_ (FUJIFILM Wako Pure Chemical Corporation, 091-07251, assay: 97%) were each weighed to a total weight of 0.5 g at a molar ratio of 2:1 and carefully ground and mixed in a mortar. The mixed powder was placed in a glass test tube that was ozonated for 15 min, and the inside of the test tube was evacuated to a pressure of *P* < 10^–1^ Pa by a diffusion pump. The glass ampoule was sealed. The glass ampoules were then wrapped in aluminum foil and placed in a tube furnace (Koyo Thermo Systems Co. Ltd., KTF035N1) for calcination. During the calcination process, the ampoules were first heated up to 150 °C at a rate of 5 °C/min, held at 150 °C for 10 h, and then cooled down to room temperature (∼20 °C) at a rate of 5 °C/min. After calcination, the ampoule was opened to obtain a dark green solid. The obtained solid was crushed into a powder in a mortar and washed with pure water (FUJIFILM Wako Pure Chemical Corporation, 161-08247) and ethanol (FUJIFILM Wako Pure Chemical Corporation, 057-00451, assay: 99.5+%) to remove the unreacted FeCl_2_. The obtained sample was placed in a desiccator and vacuum dried overnight using a diaphragm pump to remove the solvent.

### 2.2 Characterization

The XRD patterns of the powder were recorded using a diffractometer (Rigaku, Ultima IV) equipped with a Cu Kα radiation source.

FTIR spectra for samples embedded in KBr pellets were acquired using a spectrometer (JASCO Corporation, FTIR-6100).

The UV–vis absorption spectra were measured using a spectrometer (UV-1800, Shimadzu Corporation). The samples (FePc (Tokyo Chemical Industry Co. (TCI), P0774-5G, purity: >97%) or FePc(CN)_8_) were dissolved in *N*,*N*-dimethylformamide (DMF) (FUJIFILM Wako Pure Chemical Corporation, 045-02916, assay: 99.5+%), and the solution was placed in a quartz cell.

XPS (JPS-9030/JEOL Ltd.) measurements were performed using Al Kα radiation (λ = 1486.6 eV) as the excitation source. The XPS profiles presented in this study were analyzed using Voigt functions with the XPSPEAK41 software (written by Raymund W. M. Kwok).

UPS measurements were performed under an ultra-high vacuum at a base pressure of 1.0 × 10^–7^ Pa using an electron analyzer (SES200, Scienta) and a helium discharge lamp. A He Iα resonance line (*h*ν = 21.22 eV) was used as the excitation source to acquire the UPS spectra. The IPES spectra were recorded in situ on the same specimens used for the UPS measurements using a commercial apparatus (PSP Vacuum Technology). A band-pass-type photon detector for detecting photons of *h*ν = 9.3 eV, consisted of a channeltron coated with NaCl placed behind a SrF_2_ window. The Fermi level *E*_F_ was determined from the Fermi edges of Au substrates. Owing to the polymerization reaction between molecules when heated, FePc(CN)_8_ cannot be prepared vie vacuum-deposition. Consequently, the FePc(CN)_8_ samples used for the UPS and IPES measurements were prepared by dropwise addition of 40 μL of chlorobenzene onto the Au substrate, followed by the addition of 150 μL of a saturated DMF solution of FePc(CN)_8_. The samples were then dried overnight with a diaphragm pump. The Au substrates were obtained by sputtering Cr (20 nm) onto Si(100) wafers, followed by the deposition of Au (200 nm) through sputtering. The prepared Au substrates were cleaned with UV–ozone for 15 min immediately prior to use. The FePc samples were thin films prepared via vacuum evaporation on Au substrates. The thickness of the FePc film, measured using a quartz crystal microbalance, was 10 nm. The Au substrates were then obtained by the vacuum evaporation of Au on Si(100) wafers.

Electric current measurements were performed using a source-measure unit (6487 J, Keithley) and a DC power source (R6144, Advantest).

In the measurement of photocatalytic activity, FePc, FePc(CN)_8_, or phthalocyanine (H_2_Pc; Sigma-Aldrich, 253103, assay: 98%) was added to an aqueous solution of the organic dye methyl orange (MO) (Sigma-Aldrich, 114510-25G, dye content: 85%) and then stirred for 30 min to allow for the adsorption of MO onto the sample. The UV–vis absorption spectra of the suspension were then measured, and the suspension was irradiated with a Xe lamp (ASAHI SPECTRA, MAX-303, 300 W) for 5 min. The UV–vis absorption spectra of the suspension were measured again. After each 5 min of light irradiation, the UV–vis absorption measurements were repeated.

Magnetization measurements were performed using a superconducting quantum interference device (SQUID). The sample powder (approximately 10 mg) was wrapped in a cling wrap (approximately 2 × 2 cm) and placed near the center of a special straw. The masses of the samples and other materials were measured with an accuracy of 0.01 mg. Ceramic scissors were used to cut the cling wrap in which the samples were wrapped to prevent contamination with magnetic impurities. The measurements were performed after a straw containing the sample was attached to an RSO rod and inserted into the instrument. The temperature dependence of magnetization (*M*–*T*) was measured in external magnetic fields (1000 Oe), in the temperature range of 2–300 K. In the magnetic field dependence of magnetization (*M*–*H*) was measurements at temperatures of 2 K using magnetic fields ranging from −70 to 70 kOe.

### 2.3 Theoretical Calculations

The XRD profiles were calculated using Reflex/powder diffraction in the BIOVIA Materials Studio. Geometry optimization of the FePc(CN)_8_ crystal was performed using CASTEP, BIOVIA Materials Studio with functional GGA_PBE (Perdew–Burke–Ernzerhof) and pseudopotentials: OTFG ultrasoft. The FTIR simulations were performed for a single molecule using Gaussian09 (B3LYP/6-31G(d)). Optical spectra were simulated using time-dependent density functional theory (TD-DFT) with ALDA, using the Dmol3 software of BIOVIA Materials Studio. The DNP 4.4 basis set and the GGA/BLYP/TS functional were employed for the simulation. Theoretical calculations for FePc and FePc(CN)_8_ were performed for their *S* = 1 triplet ground states.

## 3 Results and Discussion

### 3.1 Crystal Structure of FePc(CN)_8_

**Figure 2:**
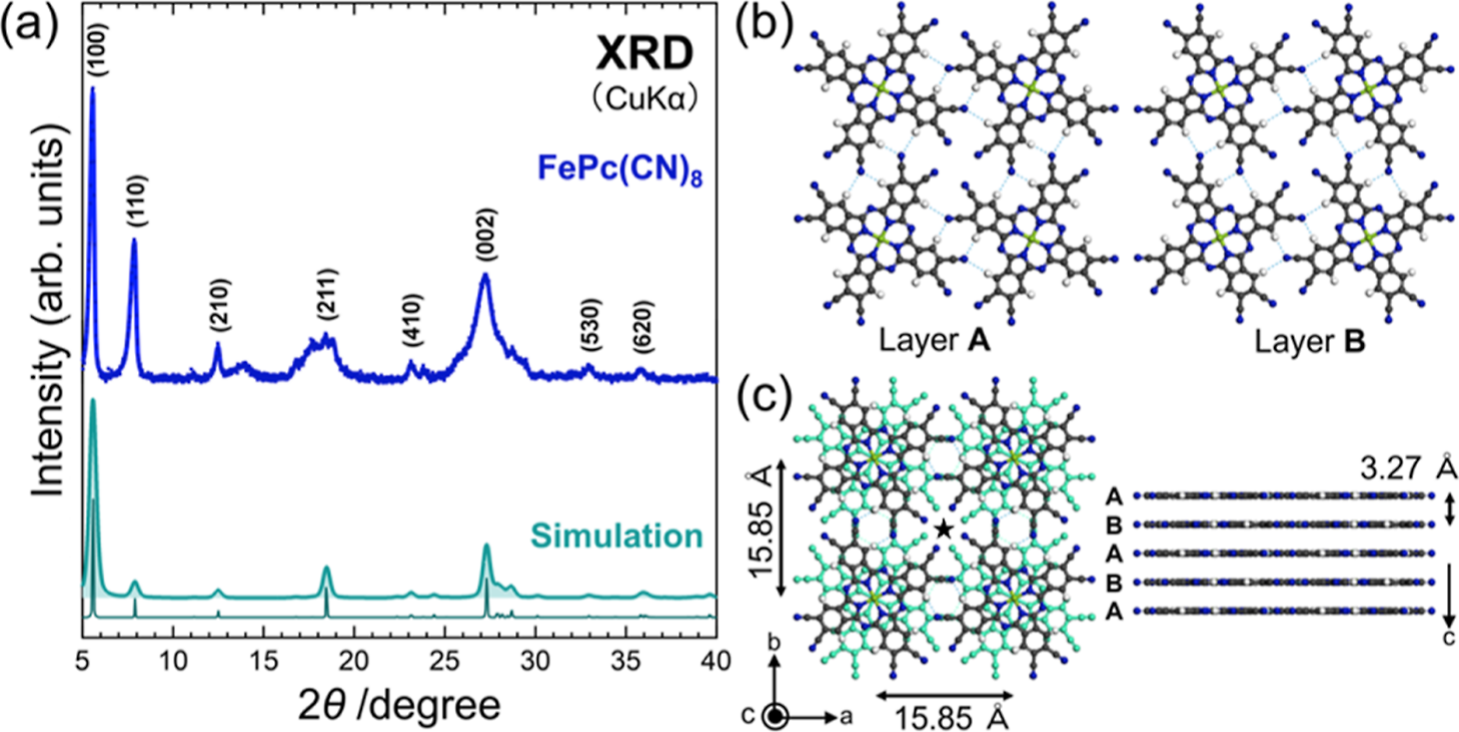
(a) XRD result of FePc(CN)_8_. The simulated XRD profiles are also shown at the bottom of the figure for a comparison. The simulated XRD profiles show two results with different line widths. (b) Structure of the molecular layers that make up the FePc(CN)_8_ crystal. Layer A has a counterclockwise rotational orientation of the molecules around the metal, while layer B has a clockwise rotational orientation. (c) Crystal structure of FePc(CN)_8_ determined by the analysis of the XRD results. The dashed lines in (b,c) represent hydrogen bonds.

Figure 2a shows a comparison of the observed and simulated XRD profiles of FePc(CN)_8_. Despite the discrepancy in the intensity ratios, the observed diffraction patterns are well explained by the simulation results. On the other hand, the intensity ratio of the (100) and (110) diffraction peaks and the peaks near (210) and (211) that cannot be explained by the results of theoretical calculations are thought to be due to polymorphs obtained in atmospheric pressure calcinations. In fact, samples synthesized in a nitrogen atmosphere at ambient pressure show a different crystal structure than those synthesized under vacuum (Figures S1–S3 in the Supporting Information). The crystal structure of FePc(CN)_8_ was strongly influenced by the terminal cyano groups of the molecules. Moreover, the neighboring FePc(CN)_8_ molecules were coupled by multiple hydrogen bonds to form layers with mirror symmetry relationships, represented as A and B in Figure 2b. The thin dashed lines in Figure 2b,c represent the hydrogen bonds between the nitrogen atom of the terminal cyano group and the hydrogen atom of the adjacent molecule. As depicted in Figure 2c, layers A and B were alternately stacked, with the Fe molecular centers aligned at the top and bottom. The FePc(CN)_8_ crystal was determined to assume tetragonal symmetry (*P*4/*mcc*) with lattice parameters *a* = *b* = 1.585 nm and *c* = 0.654 nm. The atomic coordinates are listed in Table S1 in the Supporting Information. This crystal structure, consisting of alternating stacking, is very different from the α- and β-types of monoclinic polymorphs, exhibited by many MPcs. The rotated molecules were stacked directly beneath the molecule and had a narrow one-dimensional (1D) channel in the *c*-axis direction at the location indicated by the star in Figure 2c. The thin films of LiPc exhibit either the α-form or the tetragonal x-form.^20^ The structure of the x-form consists of a 1D π-stacking parallel to the substrate, in which the molecular planes are perpendicular to the stacking axis.^21^ The x-form of LiPc is similar to the crystal structure of FePc(CN)_8_ shown in Figure 2c, although the lattice constant in the *c*-axis direction is smaller than that of FePc(CN)_8_.^20^ As an example, a similar crystal structure was reported for K_2_H_2_Pc(CN)_8_, which has K intercalated between A and B layers, formed by hydrogen bonds between H_2_Pc(CN)_8_. K is placed at the position where it forms a coordination bond with the cyano groups.^22^ Scanning electron microscopy (SEM)/ energy dispersive X-ray spectroscopy (EDX) images (Figure S4 in the Supporting Information) of a powder sample of FePc(CN)_8_ show that the sample consists mainly of angular grains with a size of 50–100 μm.

### 3.2 Chemical and Electronic States of FePc(CN)_8_

**Figure 3:**
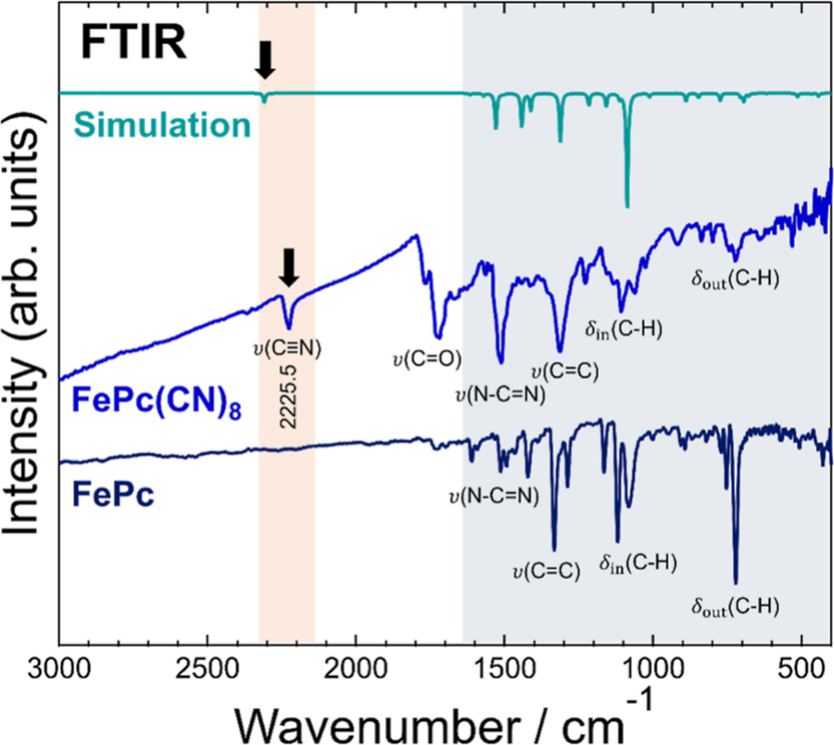
FTIR results of FePc(CN)_8_ and FePc. The red and blue shaded regions of wavenumber represent the CN stretching vibration and fingerprint region of the phthalocyanine backbone, respectively. The simulated FTIR spectrum is shown at the top. The simulated result was shifted by approximately −43 cm^–1^ to better explain the observed absorption peaks in the fingerprint region.

Figure 3 shows the FTIR spectra of FePc(CN)_8_ and FePc (detailed assignments of the absorption peaks are provided in Table S2 in the Supporting Information). In the spectra of FePc(CN)_8_, the absorptions due to the CH bending vibrations δ_in_(C–H) and δ_out_(C–H) were smaller, and the absorption due to the ν(N–C=N) stretching vibration was larger than that of FePc. The reason for the smaller absorption due to δ_in_(C–H) and δ_out_(C–H) is that the number of C–H groups in FePc(CN)_8_ is half that in FePc. The spectrum of FePc(CN)_8_ shows a cyano group stretching vibration ν(C≡N) at 2225.5 cm^–1^ that is not present in FePc, indicating the presence of cyano groups in FePc(CN)_8_. As indicated by the arrows in Figure 3, the wavenumber of the measured ν(C≡N) peak is approximately 80 cm^–1^ smaller than that of the ν(C≡N) peak in the simulated spectrum. This discrepancy is because of hydrogen bonding; in the crystal, the FePc(CN)_8_ molecules are hydrogen bonded to each other via terminal cyano groups, as shown in Figure 2b. Therefore, the bond length between the carbon and nitrogen of the cyano group is somewhat longer than that in the case of isolated molecules, and ν(C≡N) shifts to the low wavenumber side.^23^ The spectra in the fingerprint region of FePc(CN)_8_ and FePc are characteristic of phthalocyanine, indicating that FePc(CN)_8_ is a phthalocyanine molecule with cyano groups. Absorption due to the C=O stretching vibration ν(C=O), was observed at approximately 1700 cm^–1^ only in FePc(CN)_8_. The absorption due to the vibration ν(C=O) did not appear in the simulated spectrum of FePc(CN)_8_. This was caused by the hydrolysis of the cyano groups of FePc(CN)_8_ during synthesis, resulting in the formation of amides (possible product structures and their simulated IR spectra are shown in Figure S5 in the Supporting Information). A certain amount of such impurities is contained in FePc(CN)_8_, and their formation can be suppressed to some extent by lowering the humidity in the sample synthesis environment.

**Figure 4:**
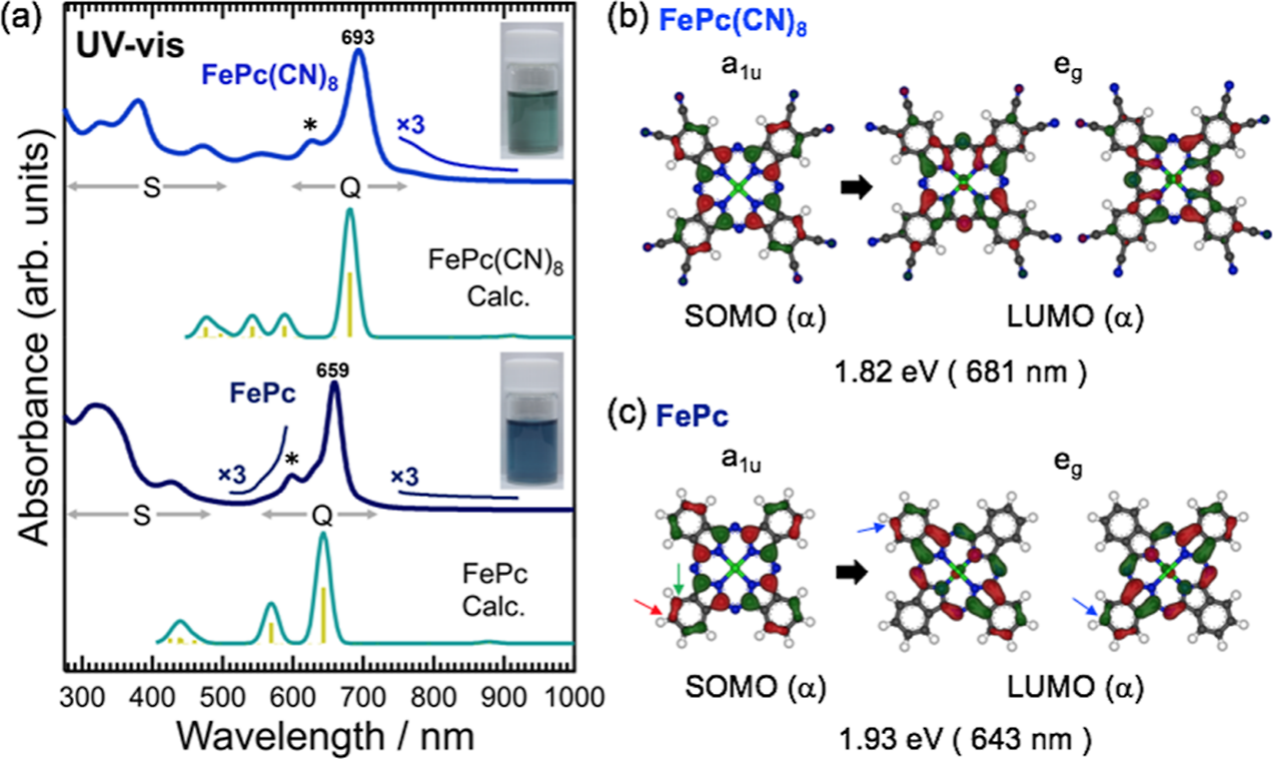
(a) UV–vis absorption spectra of FePc(CN)_8_ and FePc dissolved in DMF. The green spectra and yellow vertical bars represent the simulated UV–vis spectra of FePc(CN)_8_ Calc. and FePc Calc., respectively. The green spectra were obtained by convolving the yellow spectra with a Gauss function with a width of 20 meV. Photographs in the figure show the DMF solutions under laboratory illumination. (b,c) Optical transitions that produce Q-bands in FePc(CN)_8_ and FePc, respectively. The wavefunctions and transition energies were calculated based on DFT. The red and green parts of the wavefunction represent different signs.

Figure 4a shows the UV–vis absorption spectra of FePc(CN)_8_ and FePc. Two absorption bands, the Soret-band (S-band) and Q-band, which are characteristic of phthalocyanine, were observed for both. These absorption bands split into two in the metal-free phthalocyanine (H_2_Pc), reflecting the *D*_2*h*_ symmetry of the molecule. However, the absorption bands of MPc degenerate because of the *D*_4*h*_ symmetry of the molecule, and both the S- and Q-bands consist of a single peak. One intense peak appears at λ = 659 nm in the Q-band of FePc, as shown in Figure 4. FePc(CN)_8_ also exhibits a Q-band similar to that of FePc with a single intense peak, indicating that it has a phthalocyanine structure with Fe at the center. The absorption peaks indicated by * in Figure 4b to the left of the 693 nm peak of FePc(CN)_8_ and the 659 nm peak of FePc are caused by vibrational transitions.^24,25^ The peaks observed at 470 nm (FePc(CN)_8_) and 430 nm (FePc) may be characteristic of metal-to-ligand charge transfer (MLCT).^24,25^ The MLCT is thought to be caused by the coordination of DMF to the Fe site in FePc(CN)_8_ and FePc in the solution, resulting in a charge transfer from Fe to the *Pc* ring.^26^ FePc(CN)_8_ and FePc exhibit very similar spectra; however, the major difference is that the spectrum of FePc(CN)_8_ is redshifted by approximately 30 nm. Furthermore, the spectrum of FePc(CN)_8_ exhibits a slight absorption band up to approximately 800 nm. These differences can be observed in the color of the DMF solution, as shown in Figure 4. The FePc(CN)_8_ solution is green, whereas the FePc solution is blue. Figure 4a also shows the UV–vis spectra of the simulated FePc and FePc(CN)_8_. The simulated results agree well with the measured results. In particular, the shape and intensity of the absorption bands from 400 to 800 nm were well reproduced by the simulation. The peak around 570 nm that appears in the results of the theoretical calculations for FePc can be seen in the measured spectrum, although it is weak in intensity.

The reason for the red shift in the absorption spectrum of FePc(CN)_8_ compared to that of FePc is discussed using the Q-band as an example. The transitions in the Q-band are π → π* transitions from the α-spin singly occupied molecular orbital (SOMO) (a_1u_) to the lowest unoccupied molecular orbital (LUMO) (e_g_) for both FePc and FePc(CN)_8_, as shown in Figure 4b,c. The calculated π → π* transition energies for FePc(CN)_8_ and FePc, based on density functional theory (DFT), are 1.82 eV (681 nm) and 1.93 eV (643 nm), respectively. This difference in the transition energies was due to the difference in the energy gap between SOMO and LUMO (SOMO-LUMO gap). The calculated SOMO-LUMO gaps of α-spin orbitals for FePc(CN)_8_ and FePc were 1.393 and 1.464 eV, respectively. As shown in Figure S9 (Supporting Information), the orbital energy of FePc(CN)_8_ was significantly lower than that of FePc. This is due to the influence of the cyano groups of FePc(CN)_8_, which are strong electron-withdrawing groups. The cyano group had different effects on the SOMO and LUMO of FePc(CN)_8_. To investigate this effect, we consider the orbital energy of FePc(CN)_8_, which can be obtained by substituting the hydrogen atoms at the ends of the two FePc molecules with cyano groups. By comparing the wave function amplitude at these sites, indicated by the red arrow for the SOMO in Figure 4c and by the blue arrow for the LUMO in Figure 4c, we can see that the LUMO has a larger wave function amplitude. Therefore, the substitution of hydrogen by the cyano group had a greater impact on the LUMO. This result is evident from the fact that the SOMOs of FePc and FePc(CN)_8_ are similar, but the LUMOs differ significantly, as shown in Figure 4b. Because the LUMO was more affected by the cyano groups, the SOMO-LUMO gap of FePc(CN)_8_ was smaller than that of FePc. Similar results have been reported in previous studies. It has been reported that the Q-band of the UV–vis spectrum is red-shifted when an electron-donating group is attached to carbon, as indicated by the green arrow in Figure 4c.^27,28^ This is in contrast to the case of FePc(CN)_8_, where the red shift is caused by the destabilization of the highest occupied molecular orbital (HOMO) relative to the LUMO due to electron donation from the electron-donating group. This leads to the narrowing of the energy gap between the HOMO and LUMO orbitals and, consequently, a red shift in the Q-band transition energy.

**Figure 5:**
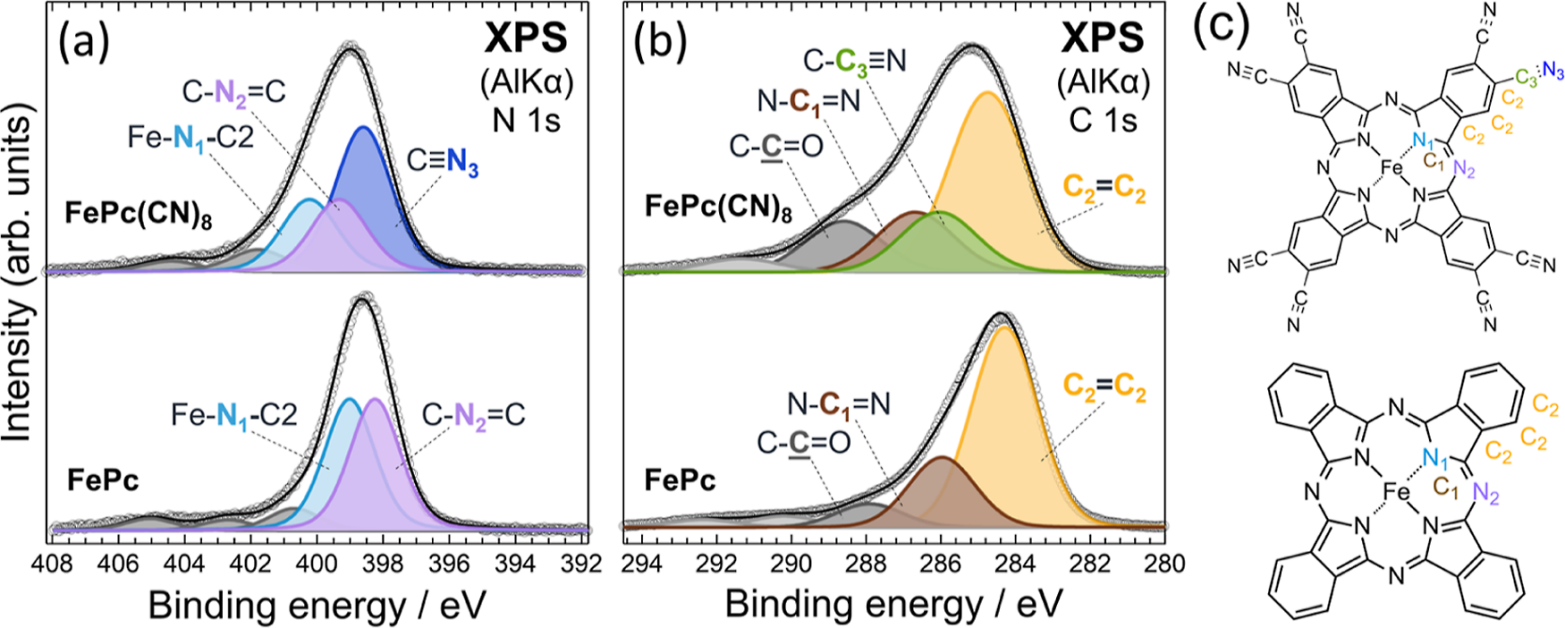
(a) N 1s XPS results of FePc(CN)_8_ and FePc. (b) C 1s XPS results of FePc(CN)_8_ and FePc. Open circles represent the measured spectra after subtracting Shirley-type backgrounds. Labels for respective peak components resolved by standard peak fitting analysis with the Voigt function correspond to the atoms labeled in the molecular structures illustrated in (c).

The relative intensity and energy of the core-level spectrum measured using XPS provide information on the chemical composition of the material. The N 1s XPS profiles of FePc(CN)_8_ and FePc are shown in Figure 5a. A fitting analysis considering three and two nitrogen atoms in different chemical environments contributed to the FePc(CN)_8_ and FePc spectra, respectively. This analysis resulted in stoichiometric XPS intensity ratios for each nitrogen atom, with N_1_:N_2_:N_3_ = 1:1:2 for FePc(CN)_8_ and N_1_:N_2_ = 1:1 for FePc. Fitting analysis considering three and two carbons in different chemical environments contributed to the FePc(CN)_8_ and FePc spectra, respectively. This analysis resulted in stoichiometric XPS intensity ratios for each carbon atom, with C_1_:C_2_:C_3_ = 1:3:1 for FePc(CN)_8_ and C_1_:C_2_ = 1:3 for FePc. However, from the stoichiometric ratios, a fitting analysis that considers the contributions of three and two carbon atoms in different chemical environments to the spectra of FePc(CN)_8_ and FePc cannot explain the spectra measured for both. Thus, the spectra were well reproduced from the stoichiometric ratios when the contribution of the carbon bonded to the oxygen in the C–C=O was also considered. That is, C_1_:C_2_:C_3_ = 1:3:1 for FePc(CN)_8_ and C_1_:C_2_ = 1:3 for FePc. Therefore, the C–C=O peak in the C 1s spectrum of FePc(CN)_8_ was due to impurities remaining in the sample, which were caused by the hydrolysis of the cyano groups during the synthetic process. This is consistent with the FTIR results shown in Figure 3. The presence of oxygen in the FePc(CN)_8_ sample was also confirmed by XPS (Figure S6 in the Supporting Information). The survey scan spectra also confirm the presence of Fe in FePc(CN)_8_. The origin of the C–C=O peak in the case of FePc has not yet been determined, but it is likely due to carbon oxide impurities. The C/N, N/Fe, and C/Fe abundance ratios of FePc(CN)_8_ estimated from the XPS results were 2.9, 15, and 43, respectively, which were close to the theoretical values of 2.5, 16, and 40, respectively. The Fe 2p XPS spectra are shown in Figure S7 in Supporting Information.

The XPS spectrum of FePc(CN)_8_ shifted toward the high-binding-energy side for both the N 1s and C 1s levels (the energies of each core level are summarized in Tables S3 and S4 in the Supporting Information). This chemical shift was due to the depletion of the electrons in the center of the FePc(CN)_8_ molecule and the effect of the electron-withdrawing cyano groups.

**Figure 6:**
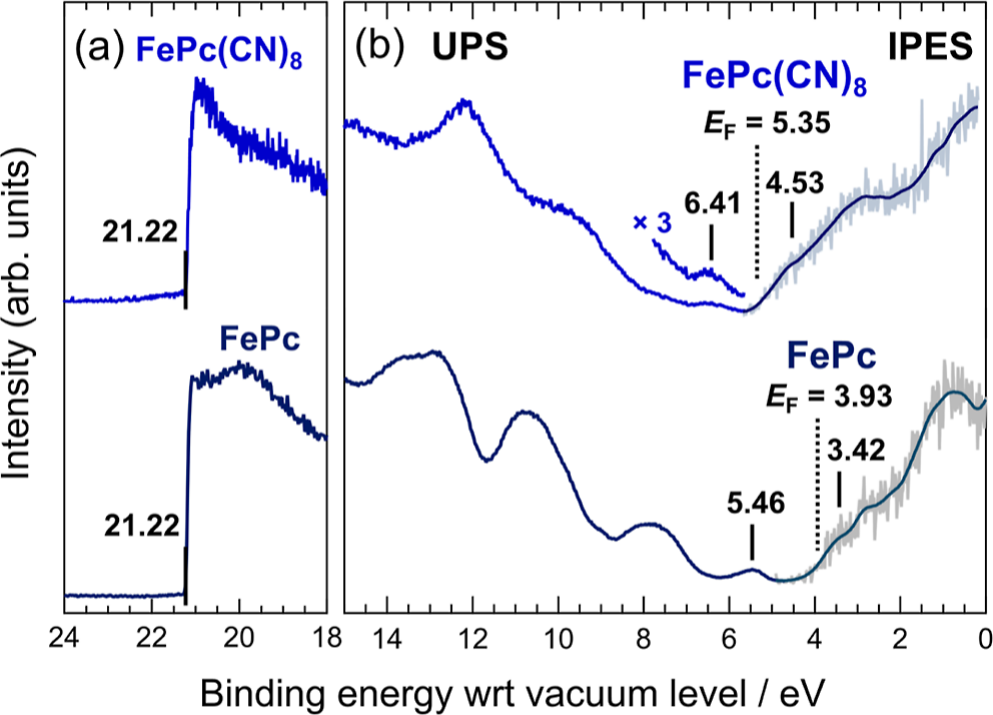
(a) UPS spectra around the secondary-electron cut-off (SECO) of FePc(CN)_8_ and FePc. Vertical lines represent the energy of SECO. (b) UPS and IPES (UPS/IPES) spectra of FePc(CN)_8_ and FePc. The bottom axis represents the binding energy measured at the vacuum level. The broken lines represent the Fermi energy (*E*_F_) of the samples. The UPS spectrum and IPES spectrum are connected at around *E*_F_. The vertical lines represent the energy of the SOMO and LUMO of the samples. The solid lines on the IPES data represent the smoothed data.

The results of the UPS/IPES measurements of FePc(CN)_8_ and FePc are shown in Figure 6. The combination of UPS and IPES provides an excellent method for probing the electronic structure around the energy gap of a molecule. The bottom axis represents the binding energy measured at the vacuum level, which can be calculated directly from the observed secondary-electron cut-off (SECO) in the UPS spectra.

The substitution of a hydrogen atom at the molecular end of phthalocyanine with an electron-withdrawing group stabilizes the molecular orbital, thus making it n-type.^28^ In a previous study, UPS/IPES measurements of CuPc, F_8_CuPc, and F_16_CuPc, in which the hydrogens at the molecular terminals were replaced with fluorines, confirmed that the HOMO and LUMO energies shift toward the higher binding energy side as the number of fluorines increases.^29^ Similarly, FePc(CN)_8_ was expected to be n-type because it has an electron-withdrawing cyano group at the molecular end.

From the UPS/IPES measurements, the SOMO and LUMO energies of FePc are 5.46 and 3.42 eV, respectively, while the SOMO and LUMO energies of FePc(CN)_8_ are 6.41 and 4.53 eV, respectively. The ionization energy and electron affinity of FePc(CN)_8_ are approximately 1 eV higher than those of FePc. By partially replacing the terminal hydrogen atoms of FePc with cyano groups, FePc(CN)_8_ became n-type. Unlike FePc, the FePc(CN)_8_ samples were not vacuum-deposited films but were prepared in air; therefore, impurities in the sample and other factors can adversely affect the accuracy of SOMO and LUMO values. According to a previous study,^29^ the electron affinities of F_8_CuPc and F_16_CuPc are 3.91 and 4.46 eV, respectively, while those of FePc(CN)_8_ are larger. The SOMO-LUMO gaps calculated from the UPS/IPES results in Figure 6 for FePc and FePc(CN)_8_ are 2.04 and 1.88 eV, respectively, with FePc(CN)_8_ having a smaller SOMO-LUMO gap. This is consistent with the UV–vis results shown in Figure 4 where the Q-band of FePc(CN)8 is observed on the longer wavelength side than that of FePc.

Based on the XRD, FTIR, UV–vis, and XPS results, it can be concluded that FePc(CN)_8_ was successfully synthesized. Furthermore, UPS/IPES measurements indicate that FePc(CN)_8_ has a strong n-type nature. In the following section, we discuss the physical properties of FePc(CN)_8_.

### 3.3 Electric Properties of FePc(CN)_8_

**Figure 7:**
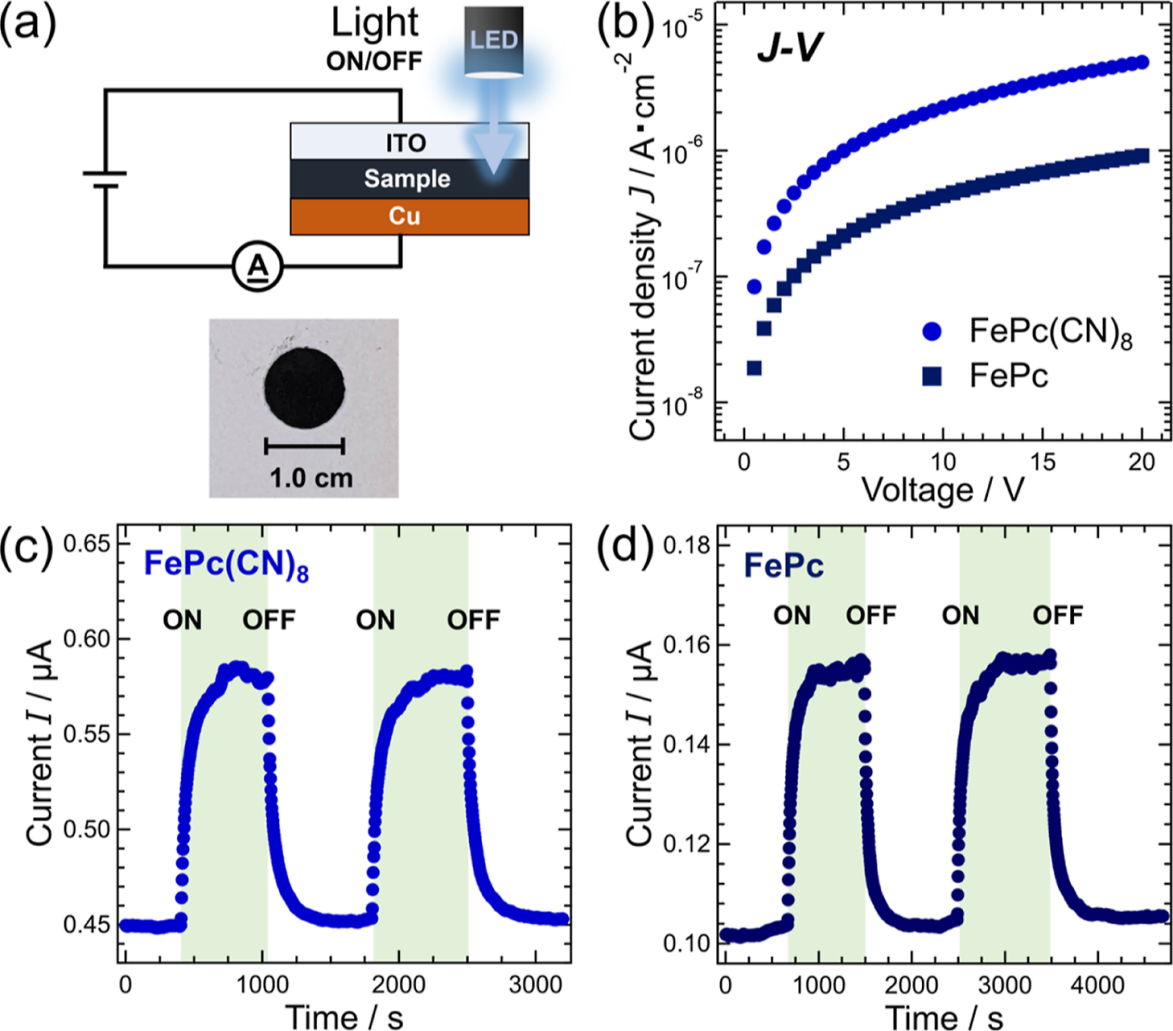
Electric properties of FePc(CN)_8_ and FePc. (a) Schematic of the measurement circuit and a photograph of a FePc(CN)_8_ pellet. The samples were pellets of FePc(CN)_8_ and FePc. (b) Results of the current–voltage (*J*–*V*) measurements of FePc(CN)_8_ and FePc. (c,d) Time-dependent current when 3 V is applied to the pellets of FePc(CN)_8_ and FePc, respectively. The green bands in the figures represent the time region where the sample was exposed to white-light.

The differences in the crystal and electronic structures of FePc(CN)_8_ and FePc lead to obvious differences in their physical properties. In this section, we discuss the differences between these two materials in terms of their electrical properties. Figure 7 illustrates the setup used to measure the electrical properties. A powdered sample of FePc(CN)_8_ or FePc was pressurized and shaped into a pellet of approximately 1 cm in diameter, which was then used as a measurement sample. Photoconduction measurements were performed by irradiating the ITO electrode with white-light from a light-emitting diode (LED). The white-light spectrum from the LED is shown in Figure S8 (Supporting Information). Figure 7b shows the results of the current–voltage (*J*–*V*) measurements of the pellet samples of FePc(CN)_8_ and FePc. The results of the electrical conductivity σ in the linear region of the low-voltage range for FePc(CN)_8_ and FePc were estimated to be σ = 4.78 ± 0.10 nS/cm and σ = 0.90 ± 0.02 nS/cm, respectively. The electrical conductivity of FePc(CN)_8_ was approximately 5.3 times greater than that of FePc. This difference in conductivity is due to the crystal structure of FePc(CN)_8_, where the molecular plane is perpendicular to the *c*-axis, resulting in a larger overlap of π-orbital than in FePc (here, α-type), where the molecular plane is inclined to the *c*-axis. Consequently, the increased overlap of π-orbitals in FePc(CN)_8_ results in higher π-electron mobility. Another possible factor is that the SOMO-LUMO gap of FePc(CN)_8_ is smaller than that of FePc, which may have reduced the charge injection barrier from the electrodes to FePc(CN)_8_.

Phthalocyanine-based molecules have been extensively studied as photoconductive materials. They have better sensitivity than other photoconductive materials at wavelengths of 780–800 nm, which is the emission wavelength of semiconductor lasers. This makes them suitable for applications such as laser printers.^30−32^ From Figure 7c,d, it was confirmed that FePc(CN)_8_ and FePc exhibit photoconductivity, as evidenced by a rapid increase in current upon light irradiation. The photocurrents increased with photoirradiation for both FePc(CN)_8_ and FePc; however, more than 200 s were required to reach saturation. Even after the photoirradiation was terminated, it took more than 500 s for the current to reach the value before photoirradiation. These behaviors are caused by charge trapping and charge accumulation by carrier traps distributed within the pellet sample and/or near the interface between the sample and electrodes. The ratio of the current increase upon light irradiation was larger for FePc (∼150%) than for FePc(CN)_8_ (∼129%). This is because the wavelength range of the white-light spectrum used for the measurement (Figure S8 in the Supporting Information) substantially overlaps with the absorption spectrum of FePc (Figure 4); thus, more excitons are produced upon white-light irradiation, resulting in a larger number of carriers obtained by charge separation.

### 3.4 Photocatalytic Activity of FePc(CN)_8_

Several MPc have long been studied for use as cocatalysts for various catalysts.^33−35^ It has also been reported that MPc itself works as a catalyst.^36−40^ MPc, similar to analogous porphyrin compounds, exhibits remarkable redox properties, and it has been used in a variety of catalysts. Additionally, it has garnered considerable attention as a photocatalyst because of its good redox activity in the photoexcited state.^37^ Here, the photocatalytic activities of FePc(CN)_8_, FePc, and H_2_Pc were evaluated based on the degradation of the organic dye MO.

**Figure 8:**
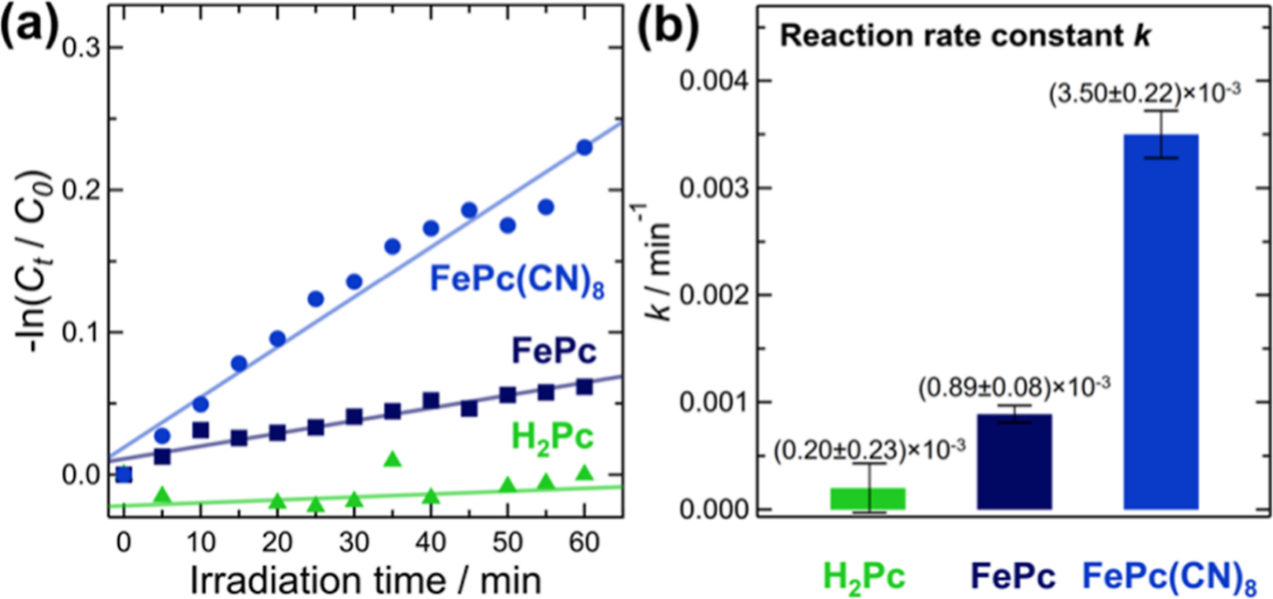
(a) Kinetic plot of the photodegradation of methyl orange (MO) by FePc(CN)_8_, FePc, and H_2_Pc under white-light irradiation. (b) Comparison of the reaction rate constants.

Figure 8 shows the measurement results for the degradation of MO dye by FePc(CN)_8_ and FePc under white-light irradiation from a Xe lamp. The reaction rate constant *k* was estimated using the following equation based on a first-order model
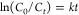
1

**Figure 9:**
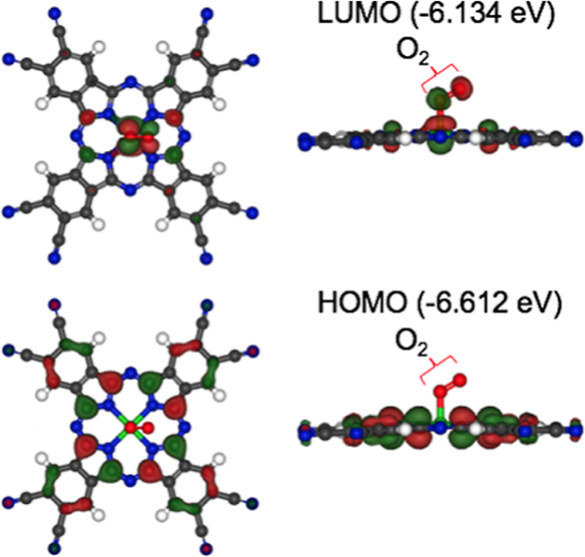
Simulated HOMO and LUMO of O_2_: FePc(CN)_8_. The numbers in parentheses represent the calculated orbital energy, measured at the vacuum level.

Here, *C*_0_ and *C*_*t*_ represent the initial equilibrium MO concentration and residual MO concentration in the solution after reaction time *t*, respectively. The deduced values of *k* are (3.50 ± 0.22) × 10^–3^ /min, (0.89 ± 0.08) × 10^–3^ /min, and (0.20 ± 0.23) × 10^–3^ /min for FePc(CN)_8_, FePc, and H_2_Pc, respectively. The value of *k* evaluated here includes the contribution of the decomposition of MO by the photocatalyst and the decrease in MO concentration due to the adsorption of MO on the sample during the measurement. Because the suspension was stirred for 30 min to maximize the adsorption of MO onto the sample before the measurements, the effect of adsorption on *k* was considered negligible. Initially, H_2_Pc exhibits little photocatalytic activity. This indicates that the Fe^2+^ in FePc and FePc(CN)_8_ affects the photocatalytic activity. The photocatalytic reaction in which MPc degrades the dye is as follows.^41^ First, MPc absorbs light and transitions to its excited state, MPc*, thereby producing an exciton. The exciton generates an electron and a hole after charge separation, and the electron reduces oxygen to produce a superoxide anion O_2_^–^, which reacts with water to produce a hydroxyl radical OH^•^ and hydroxy anion OH^–^. Meanwhile, the holes react with OH^–^ to form OH^•^. Finally, the generated OH^•^s decompose the dye. In the cases of FePc(CN)_8_ and FePc, MO is expected to be decomposed by a similar pathway. However, it is currently unclear whether FePc(CN)_8_ and FePc possess the capability to reduce O_2_. Furthermore, if FePc(CN)_8_ does produce O_2_^–^, it would be necessary to consider the role of dissolved oxygen in the mechanism by which this occurs. In other words, based on the affinity between the Fe^2+^ of FePc(CN)_8_ and oxygen molecules, we can assume that the dissolved oxygen is coordinated to FePc(CN)_8_. Figure 9 shows the HOMO and LUMO from the molecular orbital calculations when dissolved oxygen was coordinated to Fe in FePc(CN)_8_ (O_2_: FePc(CN)_8_). The molecular structure shown in Figure 9 was optimized using calculations. The HOMO is not distributed in O_2_, and the LUMO is an antibonding orbital between Fe and O_2_. Therefore, the electrons occupying the HOMO of O_2_: FePc(CN)_8_ in the ground state are expected to be excited to the LUMO after light absorption, resulting in electron transfer to O_2_ and O_2_^–^ desorption.

The results in Figure 8 show that the activity of FePc(CN)_8_ was approximately four times higher than that of FePc. This significantly greater photocatalytic activity of FePc(CN)_8_ compared to that of FePc was attributed to the difference in its electronic structure. The UV–vis spectrum shown in Figure 4 is more red-shifted for FePc(CN)_8_, possibly because of the wider distribution of visible light available in the red region. Therefore, FePc(CN)_8_ can utilize more charge-separated electrons and holes than FePc and produce more excitons upon photoexcitation. Although a quantitative comparison between the redox levels of oxygen and water and the SOMO-LUMO gap of FePc(CN)_8_ is necessary, it is plausible that the increased photocatalytic activity of FePc(CN)_8_ is due to the higher number of electrons and holes produced.

### 3.5 Magnetic Properties of FePc(CN)_8_

In general, the magnetic properties of metal complexes are strongly influenced by their crystal structures. For example, the ferromagnetic behavior of β-MnPc is attributed to its unique structure, which permits the occurrence of Mn–Mn ferromagnetic interactions.^42−44^ It is generally accepted that the *M*–*M* separation along the *b*-axis and the angle of inclination of the MPc molecular planes with respect to the crystallographic *ac* plane determine the extent of the *M*–*M* exchange interaction. In the case of x-form crystals, in which the molecular planes are stacked directly on top of each other, antiferromagnetic interactions occur between transition metal ions.^20,45−48^

**Figure 10:**
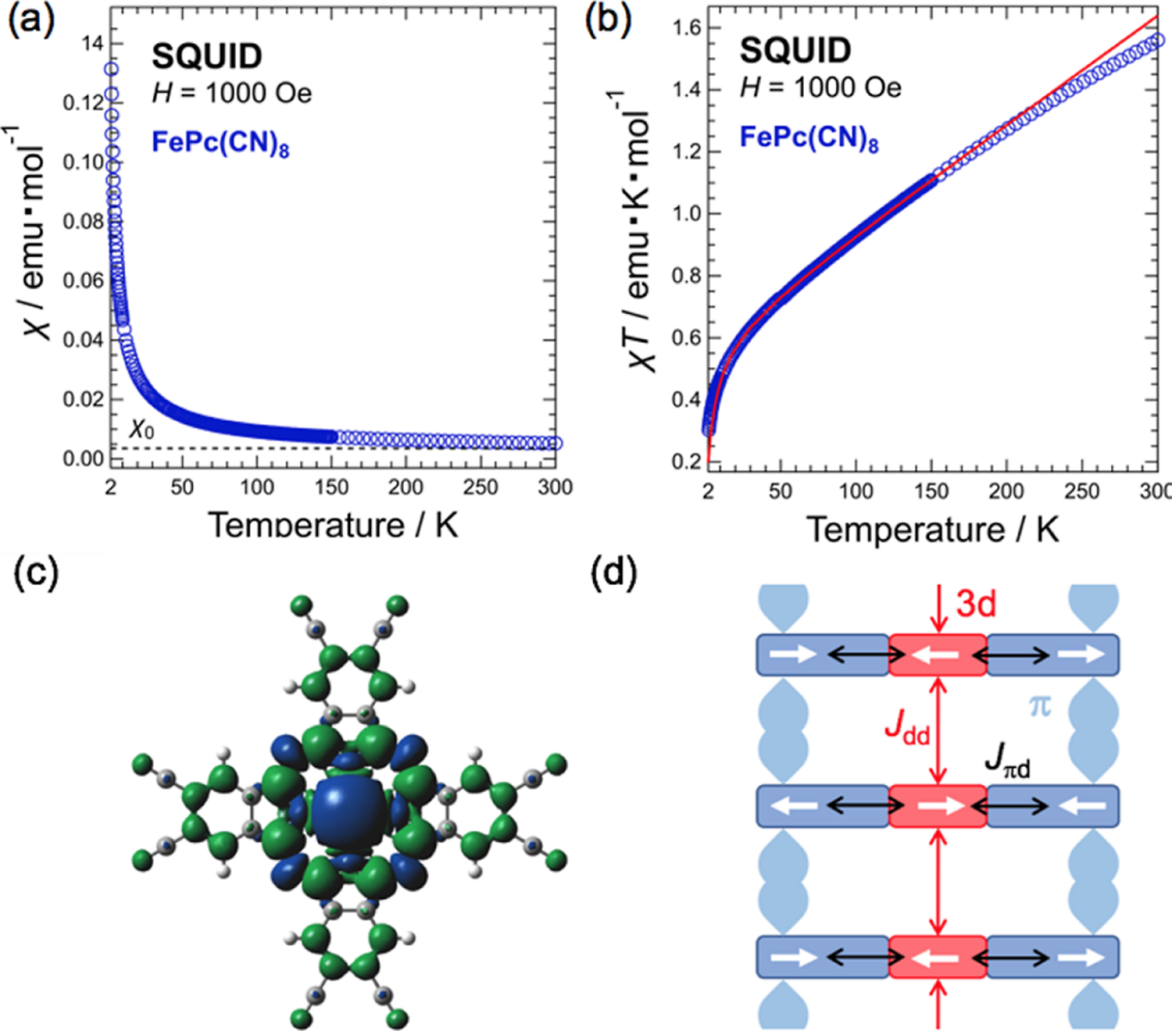
(a) Temperature dependence of thet static magnetic susceptibility χ of FePc(CN)_8_. (b) χ*T* plotted against temperature. Solid red line represents the result of the fitting analysis based on the Curie–Weiss law. (c) Calculated spin density of FePc(CN)_8_ by DFT calculation. The blue and green parts represent the density of α- and β-spins, respectively. (d) Schematic diagram of magnetic interactions between 3d orbitals and between 3d and π orbitals.

The magnetic susceptibility χ was measured by raising the temperature to 300 K while applying an external magnetic field (1000 Oe) and cooling to 2 K in the absence of an applied magnetic field (ZFC: zero-field cooling). The results of this measurement are presented in Figure 10a. As shown in Figure S10 in the Supporting Information, the dependence of magnetization (*M*) on magnetic field (*H*) (*M* vs *H*) at a temperature *T* = 2 K indicates that *M* is proportional to *H* up to an external magnetic field of 5000 Oe. Thus, when measured in an external magnetic field of 1000 Oe, the relationship between *M* and χ can be expressed as χ = *M*/*H*. χ in Figure 10 is the value obtained by subtracting the diamagnetic susceptibility calculated using the Pascal constant^49^ from the measured data. From the temperature dependence of χ in Figure 10a, FePc(CN)_8_ appears to be paramagnetic, according to Curie’s law. To better determine the magnetic interaction, χ*T* versus temperature is plotted in Figure 10b. This result shows that χ*T* is not proportional to the temperature and decreases rapidly in the low-temperature region below approximately 50 K. This behavior is different from that of paramagnetism, where χ*T* is typically proportional to temperature. The results of χ*T* indicate that antiferromagnetic interactions are dominant at low temperatures. In addition, the positive slope of χ*T* on the high-temperature side above 50 K indicates the presence of a temperature-independent component of the magnetic susceptibility, denoted as χ_0_ (χ_0_ > 0). By applying the Curie–Weiss (CW) law (χ = χ_0_ + *C*/(*T* – θ)) with the addition of the temperature-independent magnetic susceptibility term χ_0_, fitting the χ*T*–*T* result to the function χ*T* = χ_0_*T* + *CT*/(*T* – θ) (solid red line in Figure 10b) yields a Curie constant *C* = (6.025 ± 0.016) × 10^–1^ emu K mol^–1^, Weiss temperature θ = −4.3 ± 0.1 K, and temperature-independent magnetic susceptibility χ_0_ = (3.484 ± 0.011) × 10^–3^ emu mol^–1^. At high temperatures above 200 K, the χ*T* slope is different from that at lower temperatures, and there appears to be ferromagnetic interaction at high temperatures. Therefore, a fitting analysis was performed in the temperature range of 10–200 K. The reason why the χ*T* results show ferromagnetic behavior on the high-temperature side may be magnetic impurities, such as iron oxide, formed during the synthesis process. From the value of *C* for FePc(CN)_8_, the value of *S* was calculated to be *S* = 1.1. This suggests that the spin state of the Fe center is a triplet, which is consistent with the ground state of FePc being reported as *S* = 1.^50^ For the *g*-value and *C*, we used *g* = 2.100 obtained from the electron spin resonance (ESR) measurements of FePc(CN)_8_ and *C* values from SQUID measurement results (the ESR result is shown in Figure S11 in the Supporting Information).

As shown in Figure 10a,b, the magnetism of FePc(CN)_8_ consists of a nearly temperature-independent Pauli-paramagnetic term χ_0_ and a CW term, with the latter being dominant at low temperatures. The value of χ_0_ is considerably larger than that of the typical Pauli paramagnetic susceptibility. Owing to its crystal structure, the magnetic interactions in FePc(CN)_8_ have a one-dimensional ladder-like structure. This is illustrated in Figure 10c. The simulated molecular orbitals of FePc(CN)_8_ (see Figure S9 in the Supporting Information) reveal that the 3d orbitals, other than the Fe  orbital, are strongly hybridized with the *Pc* π-orbital owing to intramolecular π–d interactions. The calculated spin density shown in Figure 10c indicates that the minority β-spins are distributed on the π-orbitals of *Pc*, in contrast to the majority α-spins located on the 3d orbitals. Between neighboring molecules on the *c*-axis, the distance between the molecular planes is very short; therefore, they are coupled between the 3d orbitals of Fe^2+^ and π-orbital of *Pc*. A schematic diagram of the expected magnetic interactions is shown in Figure 10d. From the spin density calculations shown in Figure 10c, the interaction *J*_πd_ between the 3d orbital and π-orbital is positive, resulting in antiferromagnetic coupling. In such an environment, the Pauli-paramagnetism observed in the FePc(CN)_8_ magnetism can be attributed to the itinerant π-electrons of *Pc*, while the CW term is due to the antiferromagnetic coupling *J*_dd_ between the 3d orbitals of Fe^2+^. The ESR measurements of FePc(CN)_8_ show a narrow linewidth absorption with *g* = 2.004 in addition to the broad linewidth absorption of Fe^2+^. The magnetic interaction between the 3d electrons of Fe^2+^ is dominant at low temperatures, and it is probably due to the direct antiferromagnetic interactions between the 3d orbitals and the indirect interactions via the π-electrons. An example of a material that exhibits magnetic properties similar to FePc(CN)_8_ is (phthalocyaninato)cobalt hexafluoroarsenate (CoPc(AsF_6_)_0.5_).^51^ CoPc(AsF_6_)_0.5_, like FePc(CN)_8_, belongs to a tetragonal system in the *P*4/*mcc* space group, with a short distance of 3.148 Å between the molecular planes of each molecule along the *C*_4_ axis. CoPc(AsF_6_)_0.5_ is a π–d electron system that exhibits antiferromagnetic interactions between Co^2+^ and Pauli-paramagnetism owing to the π-electrons of *Pc*. The Weiss-temperature determined from the magnetic susceptibility of CoPc(AsF_6_)_0.5_ is θ = −5.2 ± 0.1 K.^51^

## 4 Conclusions

Powdered FePc(CN)_8_ was synthesized by heating (150 °C for 10 h) a mixture of TCNB and FeCl_2_ as a precursor in a vacuum glass ampoule. The crystal structure and chemical and electronic states of the obtained FePc(CN)_8_ were investigated by XRD, FTIR spectroscopy, UV–vis, XPS, and UPS/IPES measurements, and theoretical calculations in comparison with FePc. In the crystal of FePc(CN)_8_, hydrogen bonds are formed between the nitrogen and hydrogen atoms of the terminal cyano groups of adjacent molecules. As a result, the crystal structure of FePc(CN)_8_, determined by XRD, is very similar to that of the x-form of LiPc, in which the Fe at the molecular center is arranged in a one-dimensional form. UV–vis absorption measurements and theoretical calculations indicated that the LUMO of FePc(CN)_8_ was greatly affected by the terminal cyano group, resulting in the narrowing of the SOMO-LUMO gap and a red shift of the absorption spectrum, compared to FePc. Electrical measurements showed that the conductivity of FePc(CN)_8_ was approximately five times higher than that of FePc. This is because the crystal structure of FePc(CN)_8_ has smaller molecular distances in the stacking direction, which is the conduction direction, and the overlap of π-orbitals is larger than that of FePc. Similar to FePc, FePc(CN)_8_ exhibits photoconductivity. The photocatalytic activity of FePc(CN)_8_, evaluated by the decomposition of MO, was approximately four times higher than that of FePc, possibly because of the red shift in the absorption spectrum of FePc(CN)_8_ and the higher amount of light available in the visible region. The unique crystal structure of FePc(CN)_8_ also affected its magnetic properties. FePc(CN)_8_ exhibits antiferromagnetic interaction below θ = −4.3 ± 0.1 K, due to the strong intramolecular coupling between the π-orbital and Fe 3d orbital, as well as the direct intermolecular π–*d* and *d*–*d* exchange interactions. Although the FTIR and XPS results show the presence of some impurities that may be due to the hydrolysis of the cyano groups, this study established a simple method for the synthesis of FePc(CN)_8_.

FePc(CN)_8_ is a new n-type organic semiconductor expected to be used in future applications because of its wide visible light absorption band, high photocatalytic activity, and high electrical conductivity.

## Supporting Information Available

XRD results of FePc(CN)_8_ synthesized in ampoule and under atmospheric pressure in a nitrogen atmosphere, FTIR measurement results of FePc(CN)_8_ synthesized in ampoule and under atmospheric pressure in a nitrogen atmosphere, UV–vis spectra of FePc(CN)_8_ synthesized in ampoule and under atmospheric pressure in a nitrogen atmosphere, atomic coordinates of FePc(CN)_8_ crystal determined by the XRD simulation, SEM/EDX images of the powder sample of FePc(CN)_8_, assignment of FTIR spectra of FePc(CN)_8_ and FePc, FTIR spectrum of FePc(CN)_8_ and simulated FTIR spectrum of impurity molecule that may be present in the FePc(CN)_8_ product, XPS survey scan of FePc(CN)_8_ and FePc, Fe 2p XPS results for FePc(CN)_8_ and FePc, binding energies of N 1s levels of FePc(CN)_8_ and FePc determined by the analysis of XPS results, binding energies of C 1s levels of FePc(CN)_8_ and FePc determined by the analysis of XPS results, white-light spectrum of the LED used to measure the electrical characteristics, simulated MOs of FePc(CN)_8_ and FePc based on DFT, magnetic field dependence of magnetization at 2.0 K for powder sample of FePc(CN)_8_, and ESR result of FePc(CN)_8_ (PDF)
