# Supplementary material for: Synthesis and Characterization of Octacyano-Fe-Phthalocyanine
Source: ACS Omega. 2023 Jul 17;8(30):27264–75. doi: 10.1021/acsomega.3c02638 (PMC10399171; doi:10.1021/acsomega.3c02638)
Supplement: Supplementary file 1 — ao3c02638_si_001.pdf [file ao3c02638_si_001.pdf]

Supporting Information

Synthesis and characterization of octacyano-Fe-phthalocyanine

Momoka Isobe, Shota Nakayama, Shunsuke Takagi, Kakeru Araki, Kaname Kanai[*]

Department of Physics *and Astronomy*, Faculty of Science and Technology, Tokyo University of Science 2641 Yamazaki, Noda, Chiba 278-8510, Japan.

Experimental

The osmium-coated samples were used for scanning electron microscopy (SEM; FE-SEM SUPRA40; Carl Zeiss). The osmium coating was performed with a coater (Neoc-Pro; MEIWAFOSIS, Ltd.), using osmium (VIII) oxide (purity: 99.8%; FUJIFILM Wako Pure Chem. Co., Ltd.; 157-00404).

Fourier transform infrared (FTIR) spectra for samples embedded in KBr pellets were acquired using a spectrometer (JASCO Corporation, FTIR-6100).

The powder XRD patterns were recorded using a diffractometer (Rigaku, Ultima IV) equipped with a Cu-Kα radiation source.

X-ray photoemission spectroscopy (XPS) (JPS-9030/JEOL Ltd.) measurements were performed using Al Kα radiation (λ = 1486.6 eV) as the excitation source. The XPS profiles presented in this study were analyzed using Voigt functions with the XPSPEAK41 software (written by Raymund W. M. Kwok).

The PL spectrum of the LED was measured using a BIM-6002A-10 spectrometer (Brolight Technology Co., Ltd.) connected to an optical fiber.

Electron spin resonance (ESR) measurements were performed using a Bruker EMX-nano. Measurements were performed at room temperature (~ 20°C) using microwaves at the X-band frequency. Samples were measured in NMR 5 mm sample tubes NES-600 / OPTIMA.

Theoretical

The XRD profiles were calculated using Reflex/powder diffraction in BIOVIA Materials Studio. Geometry optimization of the $FePc(CN)_8$ crystal was performed using CASTEP, BIOVIA Materials Studio with functional GGA_PBE (Perdew-Burke-Ernzerhof) and pseudopotentials: OTFG ultrasoft. The FTIR simulations were performed for a single molecule using Gaussian09 (B3LYP/6-31G(d)). Molecular orbitals (MOs) were simulated using BIOVIA Materials Studio: Dmol^3 with the basis set: DNP 4.4 and functional GGA/PBE/TS. Theoretical calculations for FePc and $FePc(CN)_8$ were performed for their $S = 1$ triplet ground states.

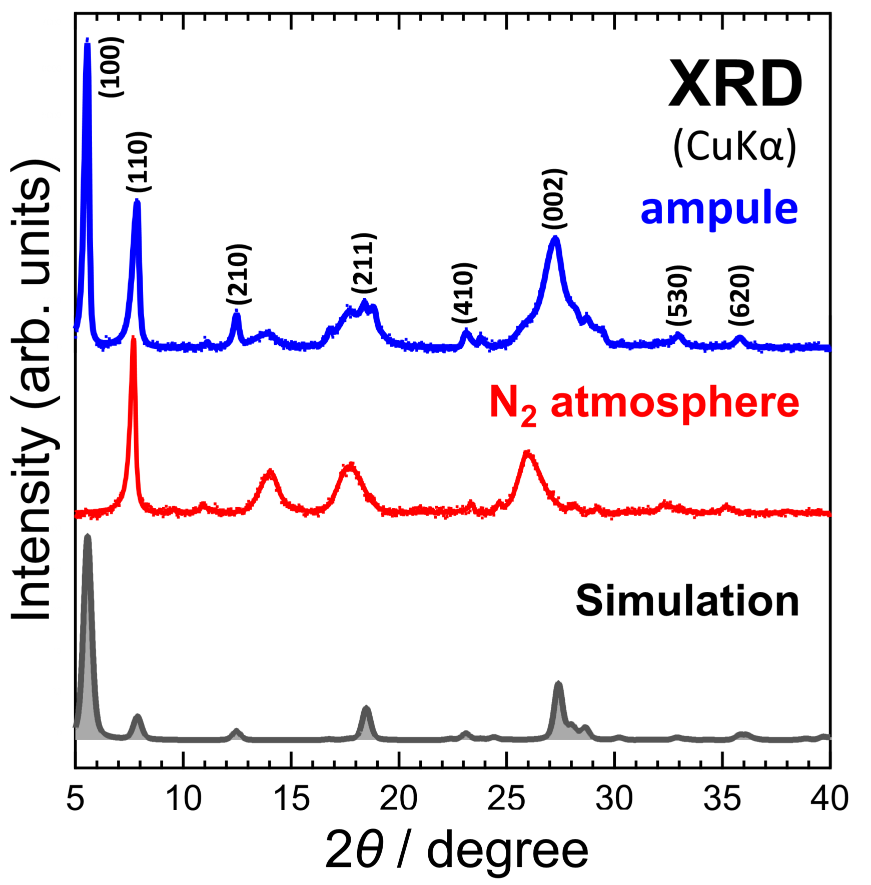

Figure S1. The blue and red lines show the XRD results of $FePc(CN)_8$ synthesized in ampoule and under atmospheric pressure in a nitrogen atmosphere, respectively. The solid black line at the bottom of the figure is the diffraction pattern obtained from the simulation for the crystal structure of $FePc(CN)_8$ synthesized in ampoule.

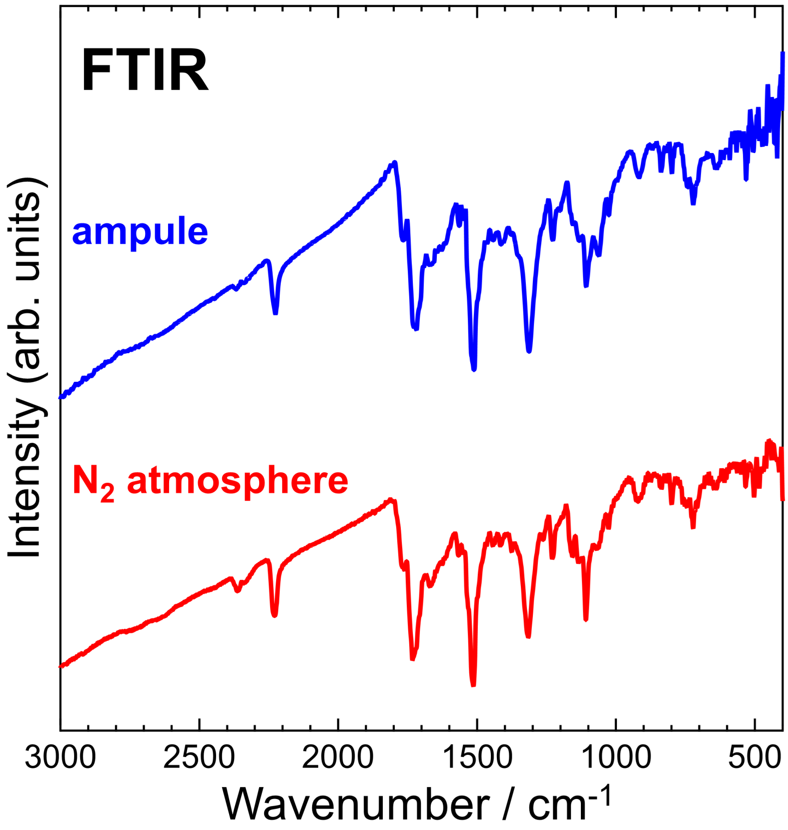

Figure S2. FTIR measurement results of $FePc(CN)_8$. The solid blue line represents the FTIR spectrum of $FePc(CN)_8$ synthesized in ampoule and the solid red line represents the FTIR spectrum of $FePc(CN)_8$ synthesized under atmospheric pressure in a nitrogen atmosphere.

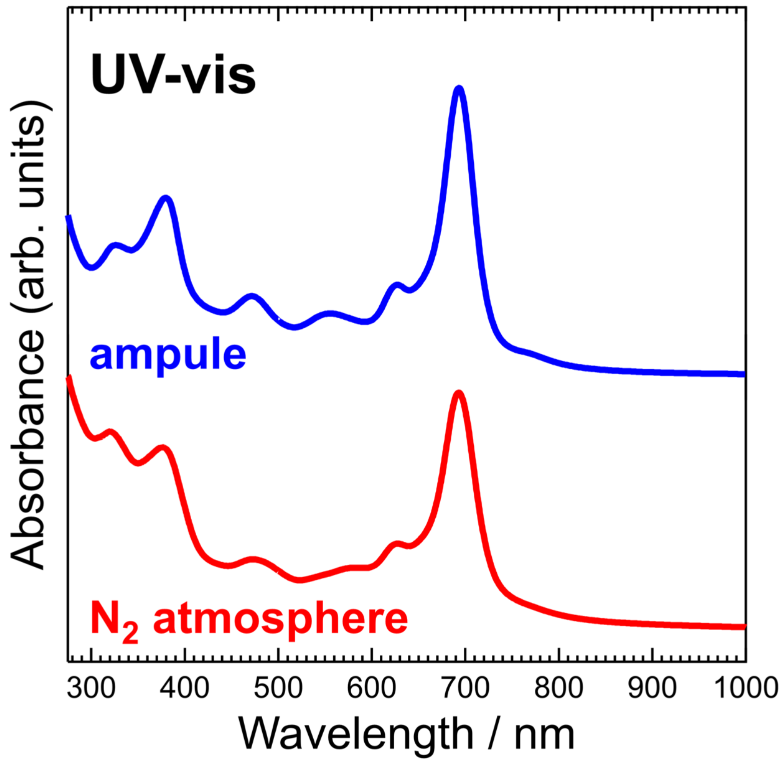

Figure S3. UV-vis spectra of $FePc(CN)_8$ in N, N-dimethylformamide solution. The solid blue line represents the spectrum of $FePc(CN)_8$ synthesized in ampoule and the solid red line represents the spectrum of $FePc(CN)_8$ synthesized under atmospheric pressure in a nitrogen atmosphere.

Table S1. Atomic coordinates of $FePc(CN)_8$ crystal determined by the XRD simulation.

Unit cell parameters: $a = b = 1.585$ nm, $c = 0.654$ nm, and $\alpha = \beta = \gamma = 90°$.

Atom	*a*	*b*	*c*
Fe1	0	0	0.5
Fe2	0	0	0
N3	0.10913	0.05107	0.5
C4	0.12598	0.13693	0.5
C5	0.21654	0.15166	0.5
C6	0.25512	0.072	0.5
C7	0.18668	0.01052	0.5
N8	-0.2002	0.07182	0.5
C9	-0.26237	-0.22614	0.5
C10	-0.35078	-0.2194	0.5
C11	-0.39019	-0.13841	0.5
C12	-0.34239	-0.06368	0.5
C13	-0.48009	-0.13598	0.5
C14	-0.40213	-0.293	0.5

N15	-0.55379	-0.13794	0.5
N16	-0.44634	-0.35206	0.5
H17	-0.23164	-0.28785	0.5
H18	-0.37284	-0.00182	0.5
N19	-0.10913	-0.05107	0.5
C20	-0.12598	-0.13693	0.5
C21	-0.21654	-0.15166	0.5
C22	-0.25512	-0.072	0.5
C23	-0.18668	-0.01052	0.5
N24	0.2002	-0.07182	0.5
C25	0.26237	0.22614	0.5
C26	0.35078	0.2194	0.5
C27	0.39019	0.13841	0.5
C28	0.34239	0.06368	0.5
C29	0.48009	0.13598	0.5
C30	0.40213	0.293	0.5
N31	0.55379	0.13794	0.5

N32	0.44634	0.35206	0.5
H33	0.23164	0.28785	0.5
H34	0.37284	0.00182	0.5
N35	-0.05107	0.10913	0.5
C36	-0.13693	0.12598	0.5
C37	-0.15166	0.21654	0.5
C38	-0.072	0.25512	0.5
C39	-0.01052	0.18668	0.5
N40	-0.07182	-0.2002	0.5
C41	0.22614	-0.26237	0.5
C42	0.2194	-0.35078	0.5
C43	0.13841	-0.39019	0.5
C44	0.06368	-0.34239	0.5
C45	0.13598	-0.48009	0.5
C46	0.293	-0.40213	0.5
N47	0.13794	-0.55379	0.5
N48	0.35206	-0.44634	0.5

H49	0.28785	-0.23164	0.5
H50	0.00182	-0.37284	0.5
N51	0.05107	-0.10913	0.5
C52	0.13693	-0.12598	0.5
C53	0.15166	-0.21654	0.5
C54	0.072	-0.25512	0.5
C55	0.01052	-0.18668	0.5
N56	0.07182	0.2002	0.5
C57	-0.22614	0.26237	0.5
C58	-0.2194	0.35078	0.5
C59	-0.13841	0.39019	0.5
C60	-0.06368	0.34239	0.5
C61	-0.13598	0.48009	0.5
C62	-0.293	0.40213	0.5
N63	-0.13794	0.55379	0.5
N64	-0.35206	0.44634	0.5
H65	-0.28785	0.23164	0.5

H66	-0.00182	0.37284	0.5
N67	-0.10913	0.05107	0
C68	-0.12598	0.13693	0
C69	-0.21654	0.15166	0
C70	-0.25512	0.072	0
C71	-0.18668	0.01052	0
N72	0.2002	0.07182	0
C73	0.26237	-0.22614	0
C74	0.35078	-0.2194	0
C75	0.39019	-0.13841	0
C76	0.34239	-0.06368	0
C77	0.48009	-0.13598	0
C78	0.40213	-0.293	0
N79	0.55379	-0.13794	0
N80	0.44634	-0.35206	0
H81	0.23164	-0.28785	0
H82	0.37284	-0.00182	0

N83	0.10913	-0.05107	0
C84	0.12598	-0.13693	0
C85	0.21654	-0.15166	0
C86	0.25512	-0.072	0
C87	0.18668	-0.01052	0
N88	-0.2002	-0.07182	0
C89	-0.26237	0.22614	0
C90	-0.35078	0.2194	0
C91	-0.39019	0.13841	0
C92	-0.34239	0.06368	0
C93	-0.48009	0.13598	0
C94	-0.40213	0.293	0
N95	-0.55379	0.13794	0
N96	-0.44634	0.35206	0
H97	-0.23164	0.28785	0
H98	-0.37284	0.00182	0
N99	0.05107	0.10913	0

C100	0.13693	0.12598	0
C101	0.15166	0.21654	0
C102	0.072	0.25512	0
C103	0.01052	0.18668	0
N104	0.07182	-0.2002	0
C105	-0.22614	-0.26237	0
C106	-0.2194	-0.35078	0
C107	-0.13841	-0.39019	0
C108	-0.06368	-0.34239	0
C109	-0.13598	-0.48009	0
C110	-0.293	-0.40213	0
N111	-0.13794	-0.55379	0
N112	-0.35206	-0.44634	0
H113	-0.28785	-0.23164	0
H114	-0.00182	-0.37284	0
N115	-0.05107	-0.10913	0
C116	-0.13693	-0.12598	0

C117	-0.15166	-0.21654	0
C118	-0.072	-0.25512	0
C119	-0.01052	-0.18668	0
N120	-0.07182	0.2002	0
C121	0.22614	0.26237	0
C122	0.2194	0.35078	0
C123	0.13841	0.39019	0
C124	0.06368	0.34239	0
C125	0.13598	0.48009	0
C126	0.293	0.40213	0
N127	0.13794	0.55379	0
N128	0.35206	0.44634	0
H129	0.28785	0.23164	0
H130	0.00182	0.37284	0

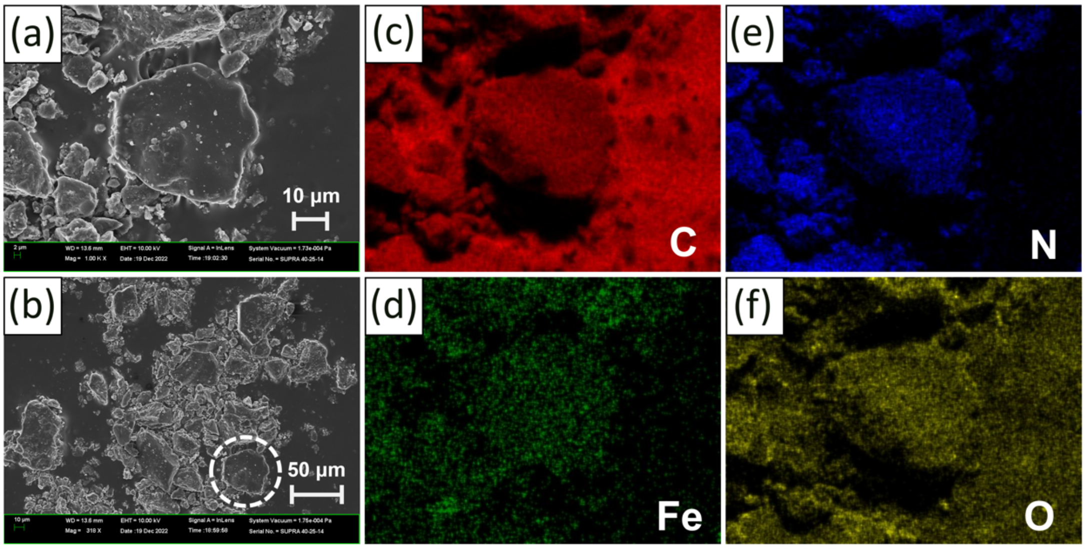

Figure S4. Scanning electron microscope (SEM)/ energy dispersive X-ray spectroscopy (EDX) images of the powder sample of $FePc(CN)_8$. (a), (b) SEM images. Distribution of (c) carbon, (d) iron, (e) nitrogen, and (f) oxygen on the image. In the mapping of carbon in (a), the red color indicating the distribution of carbon is also observed in the background, in addition to the $FePc(CN)_8$ grains because the grains are fixed on a conductive carbon tape. In the mapping of oxygen in (f), the distribution of oxygen on the grains is due to contaminants and impurities.

Table S2. Assignment of FTIR spectra of $FePc(CN)_8$ and FePc. The wavenumbers observed in Figure 3 (Exp.) and obtained by calculation (Calc.) are summarized. ν represents stretching vibration, and δ represents angular vibration. δ_{in} and δ_{out} represent in-plane and out-of-plane angular vibrations, respectively. The simulated results were shifted by approximately -43 and -33.5 cm^{-1} for $FePc(CN)_8$ and FePc, respectively, to better explain the observed absorption peaks in the fingerprint region in Figure 3.

**$FePc(CN)_8$**		
Exp. / cm^{-1}	Calc. / cm^{-1}	Assignments
637.36	696.4	ν(C=C benzene)
722.21	773.7	δ_{in}(pyrrole N-C=N)
742.46	773.7	δ_{in}(pyrrole N-C=N)
779.35	847.2	ν(pyrrole)
837.92	888.9	δ_{out}(C-H)
1025.9	1011.8	ν(isoindole N-C=N C=C)
1060.7	1086.8	δ_{in}(C-H)

1106.9	1086.8	δ_{in}(C-H)
1134.9	1112.4	δ_{in}(C-H), ν(isoindole benzene C=C)
1155.2	1157.7	δ_{in}(C-H), ν(isoindole benzene C=C)
1227.5	1216.2	ν(isoindole benzene C=C)
1313.3	1311.8	ν(isoindole benzene C=C)
1409.7	1411.5	δ_{in}(C-H)
1442.5	1441.9	δ_{in}(C-H)
1515.8	1529.1	ν(isoindole N-C=N)
1565.0	1571.2	ν(isoindole benzene C=C)
1725.0	-	ν(C=O)
1765.5	-	ν(C=O)
2225.5	2309.0	ν(C≡N)
3026.7	-	ν(O-H)

3250.1	3198.1	ν(C-H)
**FePc**		
Exp. / cm^{-1}	Calc. / cm^{-1}	Assignments
721.25	714.9	δ_{out}(C-H)
753.07	740.7	ν(isoindole)
771.39	757.2	δ_{out}(C-H)
891.92	895.0	ν(isoindole C-N=C)
907.34	895.0	ν(isoindole C-N=C)
999.91	1005.0	δ_{in}(C-H)
1081.9	1065.0	δ_{in}(C-H)
1088.6	1086.1	ν(pyrrole N) , δ_{in}(C-H)
1118.5	1123.7	ν(pyrrole N), δ_{in}(C-H)
1164.8	1169.9	ν(pyrrole N), δ_{in}(C-H)

1287.3	1298.3	δ_{in}(C-H)
1332.6	1357.0	ν(benzene C=C)
1421.3	1436.9	δ_{in}(C-H)
1493.6	1485.7	δ_{in}(C-H), ν(C-N=C)
1513.8	1528.6	ν(pyrrole C-N)
1591.0	1609.2	ν(benzene C=C)
1610.3	1629.7	ν(benzene C=C)
1726.0	3169.1	ν(C-H)
3046.0	3188.4	ν(C-H)

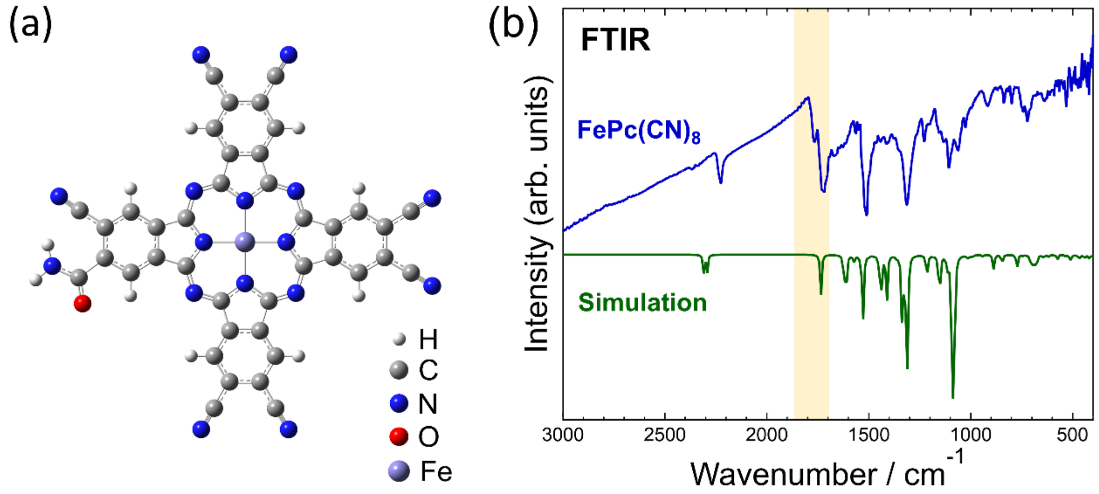

Figure S5. (a) An example of the molecular structure of impurity that may be present in the $FePc(CN)_8$ product. (b) FTIR spectrum of $FePc(CN)_8$ and simulated FTIR spectrum of the molecule in (a). The simulation was performed by density functional theory (DFT) calculation. The simulated result was shifted by approximately -43 cm^{-1} to better explain the observed absorption peaks in the fingerprint region.

It can be inferred that the impurities in the $FePc(CN)_8$ product are due to the hydrolysis of the cyano groups of TCNB or $FePc(CN)_8$ by moisture and other substances in the test tube, during the synthesis process to form amides. As shown in Figure S5(b), the simulated FTIR results of the compound in Figure S5(a) can reproduce the measured absorption peak at 1700 cm^{-1}. The compound shown in Figure S5(a) assumed a structure

containing a single amide; however, in reality, it may contain compounds with multiple amides.

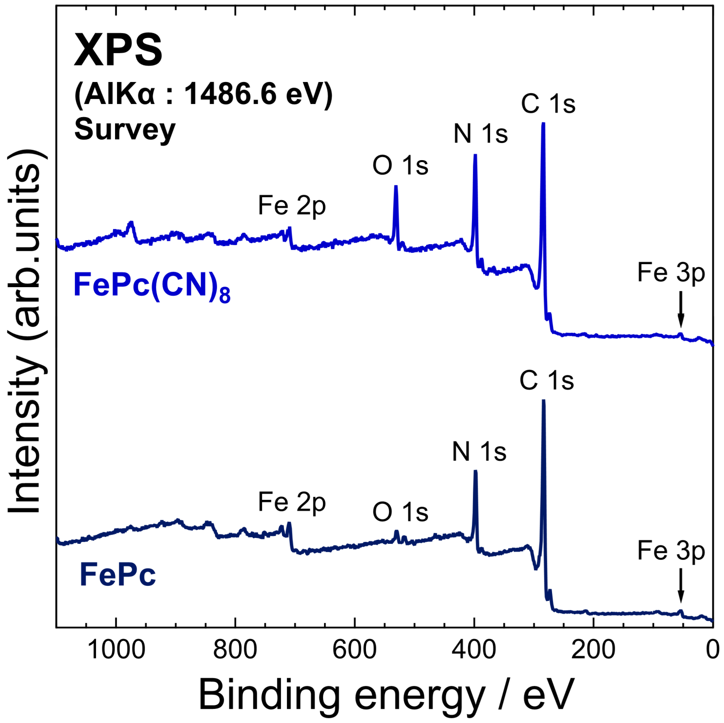

Figure S6. XPS survey scan spectra of $FePc(CN)_8$ and FePc.

Figure S6 shows the XPS survey spectra of FePc and $FePc(CN)_8$. Compared to FePc, $FePc(CN)_8$ has a higher number of nitrogen atoms, resulting in a lower C/N ratio. In both cases, the 2p and 3p levels of Fe can be seen. The large amount of oxygen in $FePc(CN)_8$

is due to the hydrolysis of some cyano groups, resulting in the formation of amides at the terminals of the molecule.

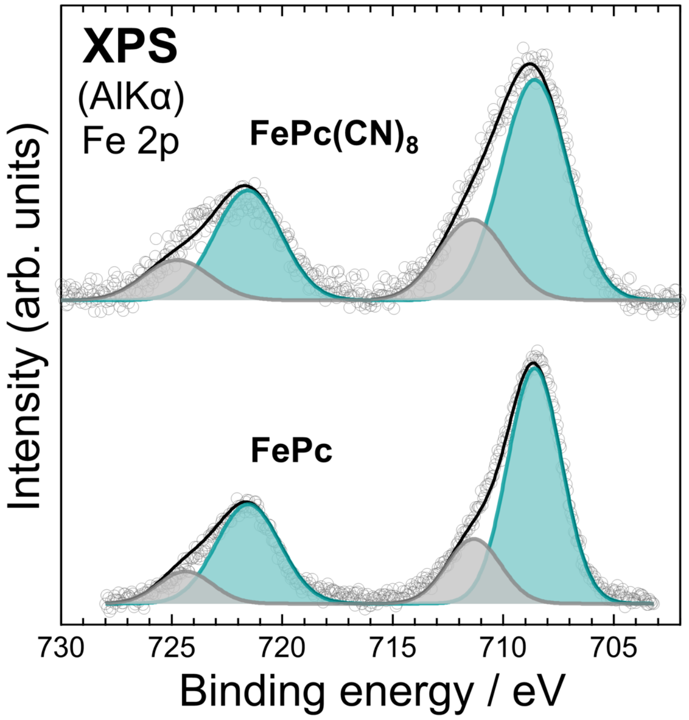

Figure S7. Fe 2p XPS results for $FePc(CN)_8$ and FePc. The circles represent the spectra with background subtracted by the Shirly method, and the black lines are the results of peak fitting analysis. The fitting was performed using the Voigt function.

Table S3. Binding energies of N 1s levels of $FePc(CN)_8$ and FePc determined by the analysis of the XPS results in Figure 5(a).

N 1s			
	$Fe-N_1-C_2$	$C=N_2-C$	$C\equiv N_3$
$FePc(CN)_8$	400.23	399.33	398.60
FePc	399.01	398.25	-

Table S4. Binding energies of C 1s levels of $FePc(CN)_8$ and FePc determined by the analysis of the XPS results in Figure 5(b).

C 1s				
	$N-C_1=N$	$C_2=C_2$	$C-C_3\equiv N$	$C-\underline{C}=O$
$FePc(CN)_8$	286.69	284.75	286.05	288.60
FePc	285.97	284.30	-	287.91

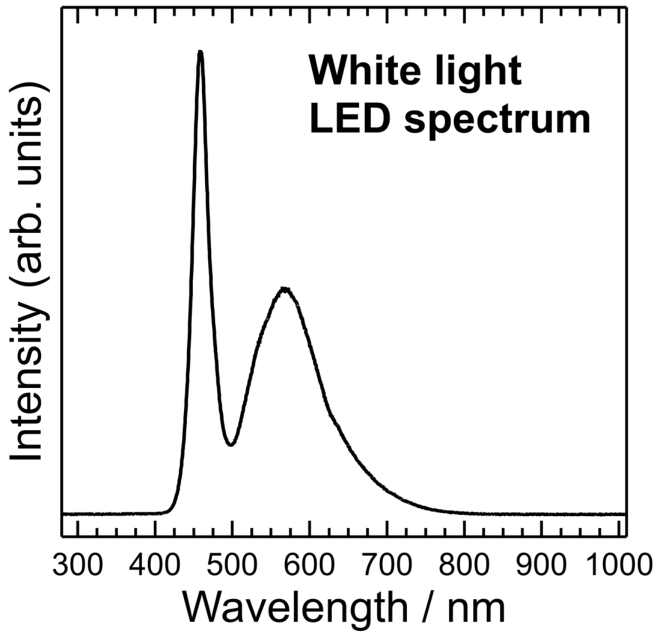

Figure S8. White-light spectrum of the LED used to measure the electrical characteristics shown in Figure 7.

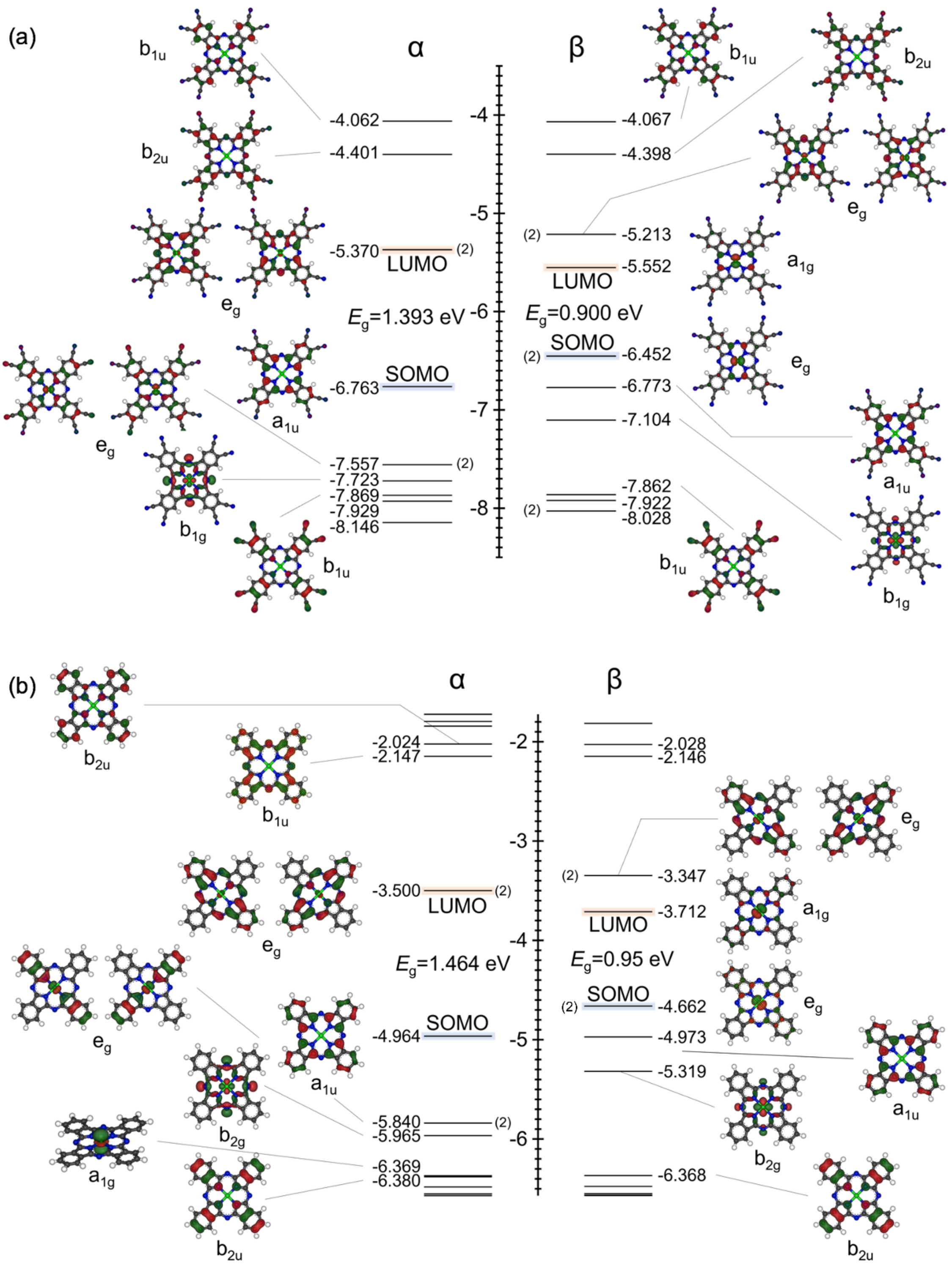

Figure S9. The simulated MOs of (a) $FePc(CN)_8$ and (b) FePc based on DFT. The

numbers in the figure are the energy of each orbital, measured from the vacuum level in eV. The red and green colors in the wavefunction represent different signs.

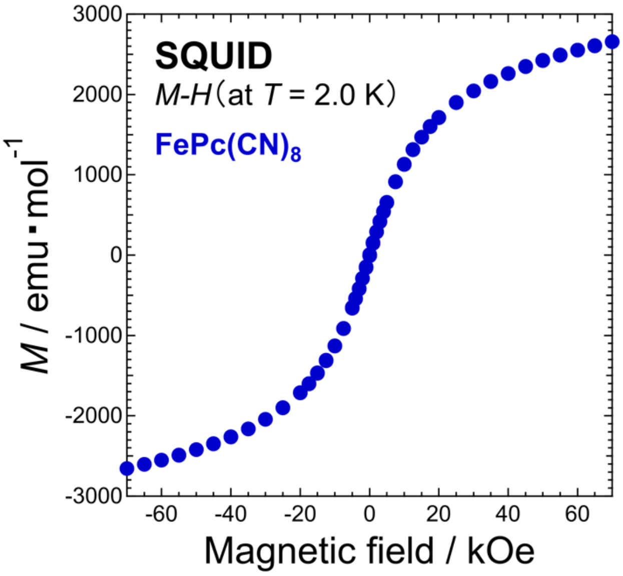

Figure S10. Magnetic field dependence of the magnetization of the $FePc(CN)_8$ powder sample at 2.0 K.

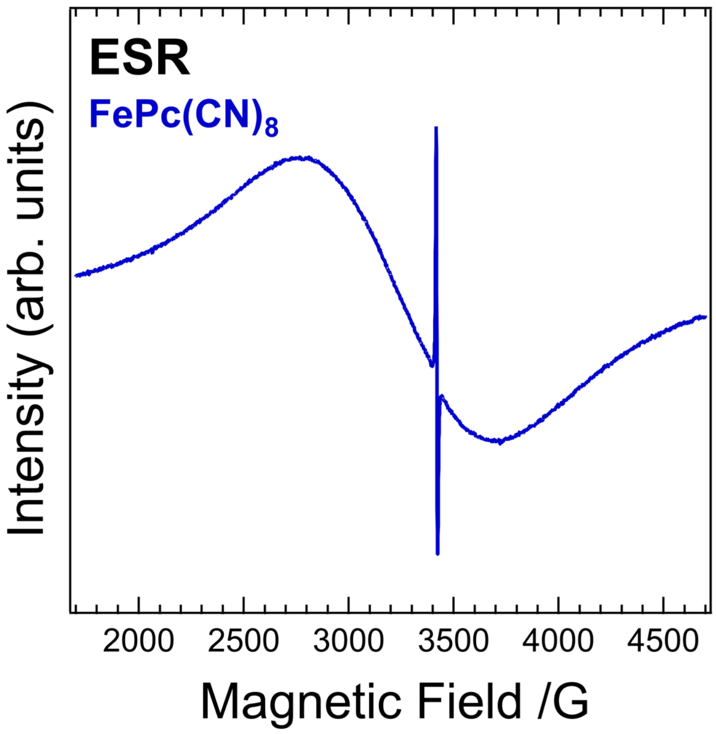

Figure S11. ESR result of $FePc(CN)_8$ measured at room temperature.
